# Supplementary material for: Species and habitat specific changes in bird activity in an urban environment during Covid 19 lockdown
Source: eLife. 2024 Feb 9;12:RP88064. doi: 10.7554/eLife.88064 (PMC10942578; doi:10.7554/eLife.88064)
Supplement: Supplementary file 1. [file elife-88064-supp1.docx]

**Supplementary file 1a. The sampling time and daily number of audio files including crow's vocalizations for each site.**

| Time | Site3 | Site4 | Site5 | Site6 | Site7 | Site8 | Site9 | Site10 | Site11 | Site12 | Site13 | Site14 | Site15 | Site16 | Site17 | Site18 | Site19 |
| --- | --- | --- | --- | --- | --- | --- | --- | --- | --- | --- | --- | --- | --- | --- | --- | --- | --- |
| 2020/3/25 | 130 | 20 | 40 | 106 | 36 | 230 | 142 | 144 | - | - | - | - | - | - | - | - | - |
| 2020/3/26 | 34 | 26 | 44 | 50 | 54 | 212 | 164 | 178 | - | - | - | - | - | - | - | - | - |
| 2020/3/27 | 18 | 14 | 38 | 60 | 36 | 264 | 180 | 190 | - | - | - | - | - | - | - | - | - |
| 2020/3/28 | 42 | 24 | 28 | 96 | 20 | 348 | 218 | 200 | - | - | - | - | - | - | - | - | - |
| 2020/3/30 | 26 | 10 | 32 | 28 | 38 | 306 | 154 | 114 | - | - | - | - | - | - | - | - | - |
| 2020/3/31 | 38 | 10 | 4 | 34 | 38 | 306 | 142 | 96 | 82 | 16 | 140 | 46 | 84 | 34 | 58 | 118 | 170 |
| 2020/4/1 | 36 | 16 | 10 | 34 | 36 | 190 | 160 | 58 | 72 | 18 | 150 | 44 | 82 | 30 | 64 | 142 | 198 |
| 2020/4/2 | 40 | 8 | 8 | 36 | 34 | 204 | 138 | 52 | 80 | 28 | 254 | 32 | 82 | 46 | 74 | 94 | 154 |
| 2020/4/3 | 18 | 16 | 16 | 64 | 26 | 238 | 168 | 152 | 74 | 64 | 302 | 30 | 88 | 58 | 111 | 162 | 230 |
| 2020/4/4 | 72 | 10 | 12 | 142 | 58 | 230 | 168 | 188 | 174 | 86 | 258 | 72 | 90 | 94 | 176 | 170 | 182 |
| 2020/4/5 | 40 | 12 | 14 | 42 | 30 | 166 | 120 | 82 | 98 | 32 | 106 | 32 | 54 | 32 | 88 | 52 | 162 |
| 2020/4/6 | 46 | 6 | 26 | 22 | 30 | 200 | 132 | 80 | 106 | 22 | 94 | 36 | 62 | 30 | 62 | 104 | 154 |
| 2020/4/7 | 62 | 8 | 30 | 48 | 36 | 180 | 160 | 90 | 84 | 24 | 104 | 40 | 36 | 32 | 44 | 78 | 146 |
| 2020/4/8 | 116 | 12 | 40 | 82 | 40 | 156 | 188 | - | 158 | 30 | 138 | 64 | 70 | 56 | 92 | 166 | 210 |
| 2020/4/9 | - | - | - | - | - | - | - | - | - | 44 | 110 | - | - | - | - | - | - |
| 2020/4/24 | - | 32 | 24 | 50 | - | 160 | 122 | 68 | 68 | 34 | - | - | - | 46 | - | - | - |
| 2020/4/25 | - | 38 | 32 | 126 | - | 210 | 160 | 124 | 80 | 42 | - | - | - | 72 | - | - | - |
| 2020/4/26 | - | 18 | 2 | 46 | - | 214 | 307 | 58 | 82 | 16 | - | - | - | 26 | - | - | - |
| 2020/4/27 | - | 44 | 8 | 44 | - | 198 | 200 | 112 | 80 | 18 | - | - | - | 24 | - | - | - |
| 2020/4/28 | - | 28 | 12 | 42 | - | 150 | 234 | 108 | 82 | 40 | - | - | - | 26 | - | - | - |
| 2020/4/29 | - | 48 | 24 | 104 | - | 208 | 510 | 242 | 186 | 100 | - | - | - | 68 | - | - | - |
| 2020/4/30 | - | 26 | 20 | 52 | - | 144 | 338 | 138 | 90 | 28 | - | - | - | 26 | - | - | - |
| 2020/5/1 | - | 48 | 44 | 62 | - | 196 | 278 | 252 | 114 | 22 | - | - | - | 18 | - | - | - |
| 2020/5/2 | - | 66 | 30 | 146 | - | 192 | 210 | 206 | 156 | 104 | - | - | - | 46 | - | - | - |
| 2020/5/3 | - | - | 20 | 94 | - | 134 | 146 | - | - | - | - | - | - | 20 | - | - | - |
| 2020/5/7 | - | 92 | 20 | 86 | - | 98 | 170 | 102 | 88 | 26 | - | - | - | 16 | - | 62 | - |
| 2020/5/8 | - | 86 | 26 | 112 | - | 130 | 192 | 102 | 134 | 28 | - | - | - | 18 | - | 74 | - |
| 2020/5/9 | - | 84 | 20 | 120 | - | 112 | 256 | 94 | 190 | 18 | - | - | - | 14 | - | 96 | - |
| 2020/5/10 | - | 60 | 20 | 88 | - | 104 | 98 | 66 | 162 | 20 | - | - | - | 22 | - | 44 | - |
| 2020/5/11 | - | 64 | 10 | 76 | - | 104 | 156 | 84 | 150 | 20 | - | - | - | 36 | - | 56 | - |
| 2020/5/12 | - | 22 | 16 | 102 | - | 106 | 390 | 78 | 142 | 10 | - | - | - | 30 | - | 84 | - |
| 2020/5/13 | - | 42 | 16 | 64 | - | 140 | 310 | 80 | 128 | 16 | - | - | - | 10 | - | 46 | - |
| 2020/5/14 | - | 72 | 10 | 82 | - | 144 | 376 | 82 | 174 | 30 | - | - | - | 14 | - | 48 | - |
| 2020/5/15 | - | 44 | 22 | 108 | - | 194 | 252 | 86 | 214 | 40 | - | - | - | 28 | - | 48 | - |
| 2020/5/16 | - | 40 | 10 | 130 | - | 144 | 238 | 76 | 136 | 70 | - | - | - | 38 | - | 62 | - |
| 2020/5/21 | 144 | 38 | 26 | 214 | 70 | 200 | 118 | 88 | 212 | 30 | 108 | 42 | 250 | 68 | 56 | 36 | 98 |
| 2020/5/22 | 150 | 60 | 54 | 222 | 70 | 186 | 168 | 120 | 198 | 36 | 160 | 42 | 268 | 38 | 110 | 32 | 152 |
| 2020/5/23 | 174 | 54 | 24 | 208 | 154 | 198 | 152 | 148 | 261 | 54 | 146 | 142 | 278 | 72 | 110 | 78 | 162 |
| 2020/5/24 | 96 | 44 | 38 | 114 | 106 | 144 | 186 | 78 | 164 | 22 | 100 | 24 | 238 | 40 | 74 | 32 | 164 |
| 2020/5/25 | 100 | 42 | 52 | 108 | 46 | 158 | 156 | 98 | 202 | 28 | 138 | 58 | 172 | 64 | 132 | 42 | 152 |
| 2020/5/26 | 78 | 68 | 44 | 104 | 62 | 176 | 134 | 74 | 194 | 44 | 116 | 60 | 172 | 34 | 118 | 42 | 134 |
| 2020/5/27 | 84 | 108 | 30 | 114 | 38 | 216 | 112 | 86 | 156 | 46 | 134 | 36 | 224 | 14 | 86 | 50 | 102 |
| 2020/5/28 | 102 | 30 | 52 | 134 | 48 | 212 | 124 | 106 | 160 | 72 | 168 | 62 | 176 | 34 | 124 | 34 | 186 |
| 2020/5/29 | 120 | 58 | 38 | - | 60 | 280 | 146 | 117 | - | 84 | 174 | 102 | 218 | 78 | 152 | 86 | 252 |
| 2020/5/30 | - | 36 | - | - | - | - | - | - | - | - | 144 | 86 | 188 | 74 | 130 | 58 | 242 |

**Supplementary file 1b. The sampling times and daily number of audio files including parakeet's vocalizations for each site.**

| Time | Site3 | Site4 | Site5 | Site6 | Site7 | Site8 | Site9 | Site10 | Site11 | Site12 | Site13 | Site14 | Site15 | Site16 | Site17 | Site18 | Site19 |
| --- | --- | --- | --- | --- | --- | --- | --- | --- | --- | --- | --- | --- | --- | --- | --- | --- | --- |
| 2020/3/25 | 10 | 46 | 104 | 14 | 20 | 150 | 10 | 78 | - | - | - | - | - | - | - | - | - |
| 2020/3/26 | 16 | 44 | 80 | 24 | 22 | 176 | 12 | 92 | - | - | - | - | - | - | - | - | - |
| 2020/3/27 | 14 | 38 | 70 | 32 | 26 | 276 | 6 | 74 | - | - | - | - | - | - | - | - | - |
| 2020/3/28 | 50 | 80 | 40 | 36 | 36 | 324 | 4 | 58 | - | - | - | - | - | - | - | - | - |
| 2020/3/30 | 6 | 48 | 46 | 38 | 20 | 166 | 10 | 26 | - | - | - | - | - | - | - | - | - |
| 2020/3/31 | 8 | 64 | 60 | 14 | 10 | 152 | 8 | 16 | 130 | 18 | 88 | 42 | 40 | 2 | 14 | 2 | 56 |
| 2020/4/1 | 18 | 70 | 20 | 18 | 18 | 154 | 2 | 16 | 118 | 12 | 84 | 46 | 34 | 0 | 4 | 6 | 64 |
| 2020/4/2 | 30 | 64 | 50 | 26 | 10 | 186 | 12 | 30 | 150 | 16 | 94 | 32 | 36 | 2 | 6 | 16 | 52 |
| 2020/4/3 | 20 | 60 | 34 | 46 | 36 | 184 | 6 | 34 | 132 | 26 | 74 | 48 | 58 | 0 | 16 | 30 | 124 |
| 2020/4/4 | 26 | 84 | 32 | 46 | 38 | 92 | 10 | 30 | 156 | 30 | 60 | 50 | 22 | 28 | 30 | 30 | 170 |
| 2020/4/5 | 12 | 54 | 24 | 46 | 2 | 102 | 10 | 36 | 102 | 4 | 84 | 28 | 36 | 2 | 10 | 14 | 82 |
| 2020/4/6 | 10 | 66 | 26 | 20 | 10 | 152 | 8 | 6 | 188 | 14 | 84 | 30 | 72 | 0 | 18 | 10 | 52 |
| 2020/4/7 | 18 | 64 | 38 | 24 | 8 | 90 | 2 | 10 | 140 | 6 | 50 | 34 | 46 | 0 | 12 | 8 | 104 |
| 2020/4/8 | 16 | 48 | 22 | 46 | 10 | 120 | 4 | - | 230 | 10 | 98 | 54 | 38 | 2 | 6 | 10 | 154 |
| 2020/4/9 | - | - | - | - | - | - | - | - | - | 28 | 76 | - | - | - | - | - | - |
| 2020/4/24 | - | 46 | 22 | 24 | - | 152 | 20 | 84 | 106 | 20 | - | - | - | 8 | - | - | - |
| 2020/4/25 | - | 50 | 20 | 46 | - | 174 | 8 | 82 | 98 | 16 | - | - | - | 8 | - | - | - |
| 2020/4/26 | - | 20 | 34 | 22 | - | 168 | 6 | 50 | 106 | 4 | - | - | - | 0 | - | - | - |
| 2020/4/27 | - | 30 | 38 | 62 | - | 182 | 10 | 66 | 114 | 2 | - | - | - | 8 | - | - | - |
| 2020/4/28 | - | 22 | 34 | 40 | - | 206 | 18 | 92 | 122 | 6 | - | - | - | 2 | - | - | - |
| 2020/4/29 | - | 40 | 42 | 50 | - | 196 | 20 | 154 | 192 | 36 | - | - | - | 12 | - | - | - |
| 2020/4/30 | - | 38 | 26 | 22 | - | 208 | 2 | 66 | 178 | 2 | - | - | - | 2 | - | - | - |
| 2020/5/1 | - | 42 | 28 | 72 | - | 176 | 18 | 106 | 138 | 18 | - | - | - | 12 | - | - | - |
| 2020/5/2 | - | 54 | 30 | 60 | - | 154 | 26 | 108 | 206 | 28 | - | - | - | 10 | - | - | - |
| 2020/5/3 | - | - | 38 | 26 | - | 148 | 20 | - | - | - | - | - | - | 0 | - | - | - |
| 2020/5/7 | - | 34 | 58 | 38 | - | 242 | 20 | 98 | 182 | 6 | - | - | - | 0 | - | 4 | - |
| 2020/5/8 | - | 40 | 64 | 86 | - | 228 | 20 | 68 | 160 | 14 | - | - | - | 2 | - | 4 | - |
| 2020/5/9 | - | 52 | 66 | 164 | - | 216 | 30 | 158 | 244 | 4 | - | - | - | 4 | - | 16 | - |
| 2020/5/10 | - | 60 | 68 | 92 | - | 250 | 32 | 78 | 150 | 6 | - | - | - | 4 | - | 4 | - |
| 2020/5/11 | - | 52 | 84 | 36 | - | 174 | 32 | 88 | 164 | 4 | - | - | - | 4 | - | 4 | - |
| 2020/5/12 | - | 34 | 72 | 52 | - | 282 | 16 | 20 | 192 | 4 | - | - | - | 4 | - | 10 | - |
| 2020/5/13 | - | 52 | 102 | 58 | - | 284 | 28 | 70 | 258 | 8 | - | - | - | 2 | - | 8 | - |
| 2020/5/14 | - | 68 | 50 | 36 | - | 272 | 12 | 60 | 224 | 6 | - | - | - | 8 | - | 16 | - |
| 2020/5/15 | - | 98 | 136 | 64 | - | 238 | 20 | 90 | 290 | 20 | - | - | - | 2 | - | 10 | - |
| 2020/5/16 | - | 86 | 52 | 32 | - | 198 | 22 | 70 | 216 | 20 | - | - | - | 14 | - | 96 | - |
| 2020/5/21 | 42 | 46 | 66 | 36 | 26 | 214 | 22 | 66 | 340 | 8 | 60 | 26 | 112 | 0 | 6 | 20 | 54 |
| 2020/5/22 | 74 | 72 | 114 | 70 | 20 | 198 | 28 | 82 | 284 | 26 | 68 | 28 | 174 | 2 | 6 | 24 | 90 |
| 2020/5/23 | 40 | 64 | 116 | 94 | 30 | 208 | 14 | 82 | 270 | 30 | 70 | 60 | 116 | 10 | 46 | 40 | 94 |
| 2020/5/24 | 20 | 40 | 70 | 64 | 26 | 180 | 8 | 52 | 150 | 12 | 28 | 28 | 72 | 4 | 2 | 12 | 64 |
| 2020/5/25 | 66 | 58 | 94 | 52 | 18 | 216 | 18 | 70 | 256 | 10 | 52 | 28 | 106 | 2 | 12 | 14 | 120 |
| 2020/5/26 | 30 | 62 | 130 | 70 | 16 | 174 | 24 | 72 | 276 | 8 | 46 | 54 | 172 | 6 | 10 | 14 | 118 |
| 2020/5/27 | 44 | 40 | 170 | 52 | 30 | 144 | 34 | 34 | 312 | 14 | 74 | 34 | 96 | 6 | 16 | 6 | 66 |
| 2020/5/28 | 42 | 44 | 126 | 60 | 34 | 156 | 14 | 64 | 314 | 20 | 58 | 26 | 90 | 0 | 12 | 14 | 100 |
| 2020/5/29 | 66 | 64 | 48 | - | 70 | 120 | 24 | 78 | - | 60 | 74 | 108 | 86 | 20 | 18 | 20 | 136 |
| 2020/5/30 | - | 68 | - | - | - | - | - | - | - | - | 108 | 100 | 90 | 32 | 6 | 22 | 110 |

**Supplementary file 1c. The sampling times and daily number of audio files including prinia's vocalizations for each site.**

| Time | Site3 | Site4 | Site5 | Site6 | Site7 | Site8 | Site9 | Site10 | Site11 | Site12 | Site13 | Site14 | Site15 | Site16 | Site17 | Site18 | Site19 |
| --- | --- | --- | --- | --- | --- | --- | --- | --- | --- | --- | --- | --- | --- | --- | --- | --- | --- |
| 2020/3/25 | 6 | 16 | 32 | 50 | 2 | 22 | 114 | 154 | - | - | - | - | - | - | - | - | - |
| 2020/3/26 | 8 | 18 | 42 | 104 | 0 | 56 | 126 | 98 | - | - | - | - | - | - | - | - | - |
| 2020/3/27 | 0 | 10 | 24 | 54 | 2 | 48 | 234 | 102 | - | - | - | - | - | - | - | - | - |
| 2020/3/28 | 18 | 12 | 42 | 50 | 6 | 28 | 100 | 90 | - | - | - | - | - | - | - | - | - |
| 2020/3/30 | 2 | 20 | 32 | 50 | 0 | 14 | 196 | 10 | - | - | - | - | - | - | - | - | - |
| 2020/3/31 | 2 | 2 | 28 | 82 | 0 | 42 | 166 | 0 | 26 | 52 | 6 | 0 | 168 | 0 | 0 | 0 | 180 |
| 2020/4/1 | 0 | 0 | 2 | 8 | 0 | 10 | 144 | 0 | 0 | 40 | 6 | 2 | 36 | 0 | 0 | 0 | 152 |
| 2020/4/2 | 0 | 2 | 2 | 22 | 4 | 6 | 190 | 2 | 20 | 144 | 4 | 2 | 126 | 0 | 0 | 2 | 70 |
| 2020/4/3 | 22 | 8 | 16 | 16 | 0 | 6 | 212 | 6 | 16 | 140 | 4 | 14 | 166 | 0 | 4 | 0 | 70 |
| 2020/4/4 | 14 | 8 | 20 | 24 | 0 | 12 | 278 | 6 | 0 | 210 | 4 | 14 | 234 | 2 | 0 | 2 | 180 |
| 2020/4/5 | 18 | 6 | 10 | 22 | 0 | 12 | 294 | 0 | 50 | 64 | 4 | 0 | 210 | 0 | 4 | 0 | 142 |
| 2020/4/6 | 10 | 14 | 54 | 18 | 0 | 14 | 178 | 6 | 6 | 22 | 0 | 2 | 246 | 0 | 12 | 0 | 62 |
| 2020/4/7 | 40 | 30 | 62 | 12 | 2 | 8 | 204 | 10 | 0 | 2 | 0 | 0 | 158 | 0 | 4 | 0 | 72 |
| 2020/4/8 | 44 | 16 | 30 | 16 | 2 | 2 | 132 | - | 6 | 6 | 12 | 8 | 96 | 0 | 6 | 0 | 110 |
| 2020/4/9 | - | - | - | - | - | - | - | - | - | 0 | 2 | - | - | - | - | - | - |
| 2020/4/24 | - | 0 | 38 | 12 | - | 74 | 360 | 138 | 16 | 2 | - | - | - | 0 | - | - | - |
| 2020/4/25 | - | 6 | 8 | 12 | - | 44 | 224 | 104 | 6 | 0 | - | - | - | 0 | - | - | - |
| 2020/4/26 | - | 4 | 10 | 14 | - | 28 | 202 | 160 | 16 | 2 | - | - | - | 0 | - | - | - |
| 2020/4/27 | - | 2 | 16 | 10 | - | 46 | 264 | 106 | 10 | 0 | - | - | - | 0 | - | - | - |
| 2020/4/28 | - | 0 | 18 | 42 | - | 98 | 198 | 26 | 8 | 2 | - | - | - | 0 | - | - | - |
| 2020/4/29 | - | 8 | 12 | 28 | - | 82 | 140 | 18 | 20 | 2 | - | - | - | 0 | - | - | - |
| 2020/4/30 | - | 0 | 22 | 14 | - | 110 | 126 | 2 | 10 | 0 | - | - | - | 0 | - | - | - |
| 2020/5/1 | - | 10 | 20 | 20 | - | 210 | 164 | 38 | 2 | 2 | - | - | - | 0 | - | - | - |
| 2020/5/2 | - | 26 | 8 | 20 | - | 226 | 216 | 28 | 4 | 2 | - | - | - | 0 | - | - | - |
| 2020/5/3 | - | - | 4 | 8 | - | 230 | 176 | - | - | - | - | - | - | 4 | - | - | - |
| 2020/5/7 | - | 0 | 2 | 2 | - | 198 | 140 | 6 | 50 | 4 | - | - | - | 0 | - | 0 | - |
| 2020/5/8 | - | 2 | 4 | 16 | - | 206 | 174 | 2 | 36 | 14 | - | - | - | 0 | - | 2 | - |
| 2020/5/9 | - | 0 | 0 | 2 | - | 156 | 152 | 0 | 30 | 8 | - | - | - | 2 | - | 0 | - |
| 2020/5/10 | - | 0 | 2 | 0 | - | 384 | 102 | 0 | 44 | 4 | - | - | - | 0 | - | 0 | - |
| 2020/5/11 | - | 0 | 2 | 6 | - | 410 | 28 | 8 | 102 | 2 | - | - | - | 0 | - | 0 | - |
| 2020/5/12 | - | 2 | 10 | 0 | - | 326 | 58 | 0 | 60 | 2 | - | - | - | 0 | - | 0 | - |
| 2020/5/13 | - | 0 | 14 | 6 | - | 294 | 66 | 0 | 36 | 6 | - | - | - | 0 | - | 2 | - |
| 2020/5/14 | - | 0 | 68 | 10 | - | 208 | 62 | 0 | 62 | 20 | - | - | - | 0 | - | 20 | - |
| 2020/5/15 | - | 0 | 68 | 10 | - | 184 | 184 | 0 | 118 | 56 | - | - | - | 0 | - | 16 | - |
| 2020/5/16 | - | 0 | 48 | 2 | - | 124 | 224 | 0 | 72 | 30 | - | - | - | 0 | - | 4 | - |
| 2020/5/21 | 0 | 22 | 0 | 22 | 24 | 144 | 178 | 0 | 10 | 0 | 4 | 10 | 138 | 0 | 2 | 0 | 68 |
| 2020/5/22 | 4 | 12 | 4 | 22 | 6 | 126 | 118 | 2 | 30 | 0 | 2 | 24 | 118 | 0 | 10 | 0 | 92 |
| 2020/5/23 | 6 | 22 | 10 | 8 | 4 | 108 | 136 | 0 | 62 | 2 | 0 | 28 | 80 | 2 | 22 | 10 | 132 |
| 2020/5/24 | 2 | 8 | 6 | 6 | 0 | 90 | 138 | 0 | 46 | 2 | 2 | 14 | 46 | 0 | 4 | 0 | 56 |
| 2020/5/25 | 2 | 14 | 2 | 64 | 0 | 92 | 104 | 0 | 20 | 0 | 2 | 20 | 34 | 2 | 0 | 0 | 34 |
| 2020/5/26 | 6 | 0 | 60 | 46 | 18 | 96 | 76 | 4 | 4 | 0 | 0 | 6 | 50 | 0 | 6 | 0 | 22 |
| 2020/5/27 | 6 | 4 | 24 | 28 | 6 | 114 | 60 | 8 | 26 | 2 | 0 | 14 | 96 | 0 | 2 | 0 | 14 |
| 2020/5/28 | 6 | 10 | 88 | 58 | 100 | 138 | 82 | 0 | 96 | 0 | 0 | 14 | 34 | 0 | 2 | 0 | 32 |
| 2020/5/29 | 0 | 12 | 30 | - | 46 | 128 | 80 | 0 | - | 4 | 0 | 20 | 10 | 0 | 6 | 0 | 82 |
| 2020/5/30 | - | 2 | - | - | - | - | - | - | - | - | 2 | 20 | 16 | 0 | 4 | 0 | 112 |

**Supplementary file 1d.** **Results from generalized linear mixed models (GLMM) comparing the differences in human activity between no lockdown period and lockdown period for all sites and for each site.**

| Dependent variable | Site category | Estimate | *P* | Percent |
| --- | --- | --- | --- | --- |
| Human_activity | All sites | -0.444 | **< 0.0001** | 35.85 |
|  | Road | 0.082 | 0.632 | 8.55 |
|  | Residence | 0.398 | **< 0.0001** | 48.88 |
|  | Park | -0.375 | **0.002** | 31.27 |

**Supplementary file 1e.** **Akaike’s information criterion (AICc) model comparison results for activity and activity variability in all species and each bird species.**

| Species | Model description | *df* | logLik | AICc | ∆AICc | *wi* |
| --- | --- | --- | --- | --- | --- | --- |
| **All species** | **Activity (Number of events/day)** ~ |  |  |  |  |  |
|  | Bird, Lock, Noise, Site, Human, Temperature, Bird*Lock, Count*Lock, Lock*Noise, Lock*Site, Lock*Human | 16 | -29807.60 | 59647.53 | 0.00 | 0.93 |
|  | Bird, Lock, Noise, Site, Human, Temperature, Bird*Lock, Count*Lock, Lock*Site, Lock*Human | 15 | -29811.28 | 59652.86 | 5.33 | 0.07 |
| **All species** | **Activity Variability (CV of the number of events/day) ~** |  |  |  |  |  |
|  | Bird, Lock, Noise, Site, Human, Temperature, Bird*Lock, Count*Lock, Lock*Noise | 14 | 235.75 | -443.21 | 0.00 | 0.22 |
|  | Bird, Lock, Noise, Site, Human, Temperature, Bird*Lock, Count*Lock, Lock*Noise, Lock*Human | 15 | 236.70 | -443.06 | 0.14 | 0.20 |
|  | Bird, Lock, Noise, Site, Human, Temperature, Bird*Lock, Lock*Noise | 13 | 234.60 | -442.96 | 0.25 | 0.19 |
|  | Bird, Lock, Noise, Site, Human, Temperature, Bird*Lock, Lock*Noise, Lock*Human | 14 | 235.53 | -442.78 | 0.43 | 0.17 |
|  | Bird, Lock, Noise, Site, Human, Temperature, Bird*Lock, Lock*Noise, Lock*Site | 15 | 235.48 | -440.62 | 2.58 | 0.06 |
|  | Bird, Lock, Noise, Site, Human, Temperature, Bird*Lock, Count*Lock, Lock*Noise, Lock*Site | 16 | 236.38 | -440.40 | 2.81 | 0.05 |
|  | Bird, Lock, Noise, Site, Temperature, Bird*Lock, Count*Lock, Lock*Noise | 13 | 233.07 | -439.89 | 3.32 | 0.04 |
|  | Bird, Lock, Noise, Site, Human, Temperature, Bird*Lock, Lock*Noise, Lock*Site, Lock*Human | 16 | 235.97 | -439.56 | 3.65 | 0.03 |
|  | Bird, Lock, Noise, Site, Human, Temperature, Bird*Lock, Count*Lock, Lock*Noise, Lock*Site, Lock*Human | 17 | 236.93 | -439.43 | 3.78 | 0.03 |
| **Hooded crow** | **Activity (Number of events/day)** ~ |  |  |  |  |  |
|  | Lock, Noise, Site, Human, Count*Lock, Lock*Site, Lock*Human | 12 | -5327.10 | 10678.79 | 0.00 | 0.48 |
|  | Lock, Noise, Site, Human, Count*Lock, Lock*Noise, Lock*Site, Lock*Human | 13 | -5326.86 | 10680.41 | 1.61 | 0.22 |
|  | Lock, Noise, Site, Human, Temperature, Count*Lock, Lock*Site, Lock*Human | 13 | -5326.88 | 10680.46 | 1.67 | 0.21 |
|  | Lock, Noise, Site, Human, Temperature, Count*Lock, Lock*Noise, Lock*Site, Lock*Human | 14 | -5326.67 | 10682.15 | 3.36 | 0.09 |
| **Hooded crow** | **Activity Variability (CV of the number of events/day) ~** |  |  |  |  |  |
|  | Lock, Noise, Site, Human, Lock*Noise, Lock*Human | 11 | 247.29 | -472.08 | 0.00 | 0.28 |
|  | Lock, Noise, Site, Human, Temperature, Lock*Noise, Lock*Human | 12 | 248.02 | -471.44 | 0.64 | 0.21 |
|  | Lock, Noise, Site, Human, Count*Lock, Lock*Noise, Lock*Human | 12 | 247.94 | -471.29 | 0.79 | 0.19 |
|  | Lock, Noise, Site, Human, Temperature, Count*Lock, Lock*Noise, Lock*Human | 13 | 248.26 | -469.83 | 2.25 | 0.09 |
|  | Lock, Noise, Site, Human, Lock*Noise, Lock*Site, Lock*Human | 13 | 247.99 | -469.29 | 2.79 | 0.07 |
|  | Lock, Noise, Site, Human, Count*Lock, Lock*Noise, Lock*Site, Lock*Human | 14 | 248.92 | -469.05 | 3.03 | 0.06 |
|  | Lock, Noise, Site, Human, Temperature, Lock*Noise, Lock*Site, Lock*Human | 14 | 248.69 | -468.58 | 3.50 | 0.05 |
|  | Lock, Noise, Human, Lock*Noise, Lock*Human | 9 | 243.29 | -468.23 | 3.85 | 0.04 |
| **Rose-ringed**  **parakeet** | **Activity (Number of events/day)** ~ |  |  |  |  |  |
|  | Lock, Noise, Site, Human, Temperature, Lock*Noise, Lock*Site, Lock*Human | 13 | -3631.41 | 7289.51 | 0.00 | 0.60 |
|  | Lock, Noise, Site, Human, Temperature, Count*Lock, Lock*Noise, Lock*Site, Lock*Human | 14 | -3630.81 | 7290.43 | 0.91 | 0.38 |
|  | Lock, Noise, Site, Human, Temperature, Count*Lock, Lock*Site, Lock*Human | 13 | -3635.75 | 7298.19 | 8.67 | 0.01 |
|  | Lock, Noise, Site, Human, Temperature, Lock*Site, Lock*Human | 12 | -3636.94 | 7298.47 | 8.96 | 0.01 |
| **Rose-ringed**  **parakeet** | **Activity Variability (CV of the number of events/day) ~** |  |  |  |  |  |
|  | Lock, Noise, Site, Human, Temperature, Count*Lock, Lock*Site, Lock*Human | 14 | 462.79 | -896.76 | 0.00 | 0.58 |
|  | Lock, Noise, Site, Human, Temperature, Count*Lock, Lock*Noise, Lock*Site, Lock*Human | 15 | 463.14 | -895.35 | 1.41 | 0.29 |
|  | Lock, Noise, Site, Human, Count*Lock, Lock*Site, Lock*Human | 13 | 460.23 | -893.76 | 3.00 | 0.13 |
| **Graceful prinia** | **Activity (Number of events/day)** ~ |  |  |  |  |  |
|  | Lock, Noise, Site, Human, Temperature, Count*Lock, Lock*Noise, Lock*Site, Lock*Human | 14 | -8002.31 | 16033.42 | 0.00 | 0.99 |
|  | Lock, Noise, Site, Human, Temperature, Count*Lock, Lock*Noise, Lock*Site | 13 | -8008.61 | 16043.92 | 10.5 | 0.01 |
| **Graceful prinia** | **Activity Variability (CV of the number of events/day) ~** |  |  |  |  |  |
|  | Lock, Noise, Site, Human, Temperature, Count*Lock, Lock*Site | 12 | -227.65 | 480.08 | 0.00 | 0.11 |
|  | Lock, Noise, Site, Temperature | 9 | -230.97 | 480.40 | 0.32 | 0.09 |
|  | Lock, Noise, Site, Temperature, Lock*Site | 11 | -228.89 | 480.44 | 0.36 | 0.09 |
|  | Lock, Noise, Site, Temperature, Count*Lock | 10 | -230.01 | 480.57 | 0.49 | 0.09 |
|  | Lock, Site, Temperature, Count*Lock, Lock*Site | 11 | -229.3 | 481.26 | 1.18 | 0.06 |
|  | Noise, Site, Temperature | 8 | -232.53 | 481.42 | 1.34 | 0.06 |
|  | Lock, Noise, Site, Temperature, Count*Lock, Lock*Noise, Lock*Site | 13 | -227.44 | 481.81 | 1.73 | 0.05 |
|  | Lock, Noise, Site, Human, Temperature, Count*Lock, Lock*Site | 13 | -227.45 | 481.83 | 1.75 | 0.05 |
|  | Lock, Site, Temperature, Count*Lock | 9 | -231.73 | 481.92 | 1.84 | 0.04 |
|  | Lock, Noise, Site, Human, Temperature, Lock*Site | 12 | -228.76 | 482.32 | 2.24 | 0.04 |
|  | Lock, Noise, Site, Temperature, Lock*Noise, Lock*Site | 12 | -228.82 | 482.43 | 2.36 | 0.03 |
|  | Lock, Site, Temperature, Lock*Site | 10 | -230.96 | 482.48 | 2.40 | 0.03 |
|  | Lock, Noise, Site, Human, Temperature | 10 | -230.97 | 482.49 | 2.41 | 0.03 |
|  | Lock, Noise, Site, Temperature, Lock*Noise | 10 | -230.97 | 482.49 | 2.42 | 0.03 |
|  | Lock, Site, Temperature | 8 | -233.12 | 482.59 | 2.52 | 0.03 |
|  | Lock, Noise, Site, Human, Temperature, Count*Lock | 11 | -229.99 | 482.64 | 2.56 | 0.03 |
|  | Lock, Noise, Site, Temperature, Count*Lock, Lock*Noise | 11 | -229.99 | 482.65 | 2.57 | 0.03 |
|  | Lock, Site, Human, Temperature, Count*Lock, Lock*Site | 12 | -229.02 | 482.83 | 2.75 | 0.03 |
|  | Lock, Noise, Site, Human, Temperature, Count*Lock, Lock*Noise, Lock*Site | 14 | -227.20 | 483.46 | 3.38 | 0.02 |
|  | Noise, Site, Human, Temperature | 9 | -232.51 | 483.48 | 3.40 | 0.02 |
|  | Lock, Noise, Site, Human, Temperature, Count*Lock, Lock*Site, Lock*Human | 14 | -227.38 | 483.82 | 3.74 | 0.02 |
|  | Lock, Site, Human, Temperature, Count*Lock | 10 | -231.69 | 483.93 | 3.85 | 0.02 |

Bird: Bird species. Lock: Lockdown status. Site: Site category. Human: Human activity. Count: Count down. AICc: Akaike’s information criterion corrected for small sample size. ΔAICc: the difference between the alternative model and best-fitting model. Models are ranked based on the AICc values from the best to the worst model. *: Interaction effect.

**Supplementary file 1f. Results from post hoc tests comparing the differences in activity and activity variability** **between no lockdown period and lockdown period in all species for each site. Estimates were calculated in % per day for the following units: Temp – per degree, Noise – per dB, Human activity – per 1 talking event, Lockdown related parameter – per existence of the lockdown (yes/no).**

| Species | Dependent variable | Site category | Estimate | *P* | Percent |
| --- | --- | --- | --- | --- | --- |
| All species | **Activity**-  Number of events/day | Road | -0.136 | 0.230 | 12.716 |
|  |  | Residence | 0.029 | 0.800 | 2.942 |
|  |  | Park | -0.043 | 0.707 | 4.209 |
|  | **Activity variability**-  CV of the number of events/day | Road | -0.037 | 0.260 | - |
|  |  | Residence | -0.016 | 0.663 | - |
|  |  | Park | -0.001 | 0.988 | - |
| Hooded crow | **Activity**-  Number of events/day | Road | 0.032 | 0.828 | 3.252 |
|  |  | Residence | 0.328 | **0.025** | 38.819 |
|  |  | Park | 0.258 | 0.076 | 29.434 |
|  | **Activity variability**-  CV of the number of events/day | Road | -0.046 | 0.217 | - |
|  |  | Residence | -0.078 | 0.080 | - |
|  |  | Park | -0.034 | 0.461 | - |
| Rose-ringed parakeet | **Activity**-  Number of events/day | Road | 0.066 | 0.664 | 6.823 |
|  |  | Residence | 0.242 | 0.114 | 27.379 |
|  |  | Park | 0.643 | **< 0.0001** | 90.218 |
|  | **Activity variability**-  CV of the number of events/day | Road | -0.029 | 0.332 | - |
|  |  | Residence | -0.070 | **0.038** | - |
|  |  | Park | -0.128 | **0.0003** | - |
| Graceful prinia | **Activity-**  Number of events/day | Road | -0.440 | 0.511 | 2.858 |
|  |  | Residence | -1.580 | **0.018** | 6.761 |
|  |  | Park | -1.640 | **0.014** | 12.015 |
|  | **Activity variability**-  CV of the number of events/day | Road | -0.038 | 0.781 | - |
|  |  | Residence | 0.142 | 0.285 | - |
|  |  | Park | 0.303 | **0.030** | - |

CV: coefficient of variance

**Supplementary file 1g. Assessment of model fit of the discriminant function analyses on the parameters of the best model in three bird species.**

| Species | Discriminant  function | Eigenvalue | Percentage variance | Test of function | Wilks’s lambda | Chi-square | *df* | *P* |
| --- | --- | --- | --- | --- | --- | --- | --- | --- |
| Hooded crows | 1 | 0.233 | 95.3 | 1-4 | 0.802 | 117.917 | 16 | < 0.001 |
|  | 2 | 0.009 | 3.8 | 2-4 | 0.989 | 6.068 | 9 | 0.733 |
|  | 3 | 0.002 | 0.7 | 3-4 | 0.998 | 1.082 | 4 | 0.897 |
|  | 4 | 0.001 | 0.2 | 4 | 1.000 | 0.001 | 1 | 0.981 |
| Rose-ringed parakeets | 1 | 0.241 | 85.9 | 1-3 | 0.775 | 136.174 | 15 | < 0.001 |
|  | 2 | 0.032 | 11.1 | 2-3 | 0.962 | 20.846 | 8 | 0.008 |
|  | 3 | 0.008 | 3.0 | 3 | 0.992 | 4.268 | 3 | 0.234 |
| Gracefull prinias | 1 | 0.065 | 77.6 | 1-4 | 0.922 | 43.441 | 20 | 0.002 |
|  | 2 | 0.012 | 14.5 | 2-4 | 0.982 | 9.918 | 12 | 0.623 |
|  | 3 | 0.007 | 7.8 | 3-4 | 0.993 | 3.494 | 6 | 0.745 |
|  | 4 | 0.001 | 0.1 | 4 | 1.000 | 0.010 | 2 | 0.995 |

**Supplementary file 1h. Akaike’s information criterion (AICc) model comparison results for activity and activity variability in all species and each bird species.**

| Species | Model description | *df* | logLik | AICc | ∆AICc | *wi* |
| --- | --- | --- | --- | --- | --- | --- |
| **All species** | **Activity (Number of syllables/day)** ~ |  |  |  |  |  |
|  | Bird, Lock, Noise, Site, Human, Temperature, Bird*Lock, Count*Lock, Lock*Noise, Lock*Site, Lock*Human | 16 | -456966 | 913964.7 | 0.00 | 1 |
|  | Bird, Lock, Noise, Site, Human, Temperature, Bird*Lock, Count*Lock, Lock*Site, Lock*Human | 15 | -457001 | 914033.2 | 68.52 | 0 |
| **All species** | **Activity Variability (CV of the number of syllables/day) ~** |  |  |  |  |  |
|  | Bird, Lock, Noise, Site, Human, Temperature, Bird*Lock, Lock*Noise, Lock*Human | 14 | 88.93 | -149.58 | 0.00 | 0.29 |
|  | Bird, Lock, Noise, Site, Human, Temperature, Bird*Lock, Count*Lock, Lock*Noise, Lock*Human | 15 | 89.59 | -148.85 | 0.73 | 0.20 |
|  | Bird, Lock, Noise, Site, Human, Temperature, Bird*Lock, Lock*Noise | 13 | 87.51 | -148.77 | 0.81 | 0.19 |
|  | Bird, Lock, Noise, Site, Human, Temperature, Bird*Lock, Count*Lock, Lock*Noise | 14 | 88.17 | -148.04 | 1.54 | 0.13 |
|  | Bird, Lock, Noise, Site, Temperature, Bird*Lock, Lock*Noise | 12 | 85.15 | -146.10 | 3.48 | 0.05 |
|  | Bird, Lock, Noise, Site, Human, Temperature, Bird*Lock, Lock*Noise, Lock*Site | 15 | 88.21 | -146.09 | 3.49 | 0.05 |
|  | Bird, Lock, Noise, Site, Human, Temperature, Bird*Lock, Count*Lock, Lock*Noise, Lock*Site, Lock*Human | 16 | 89.12 | -145.87 | 3.71 | 0.05 |
|  | Bird, Lock, Noise, Site, Temperature, Bird*Lock, Count*Lock, Lock*Noise | 13 | 85.92 | -145.59 | 3.99 | 0.04 |
| **Hooded crow** | **Activity (Number of syllables/day)** ~ |  |  |  |  |  |
|  | Lock, Noise, Site, Human, Count*Lock, Lock*Noise, Lock*Site, Lock*Human | 13 | -25425.3 | 50877.20 | 0.00 | 0.70 |
|  | Lock, Noise, Site, Human, Temperature, Count*Lock, Lock*Noise, Lock*Site, Lock*Human | 14 | -25425 | 50878.86 | 1.67 | 0.30 |
| **Hooded crow** | **Activity Variability (CV of the number of syllables/day) ~** |  |  |  |  |  |
|  | Lock, Noise, Site, Human, Temperature, Lock*Noise, Lock*Human | 12 | 184.99 | -345.40 | 0.00 | 0.16 |
|  | Lock, Noise, Site, Human, Lock*Noise, Lock*Human | 11 | 183.92 | -345.34 | 0.06 | 0.16 |
|  | Lock, Noise, Site, Human, Lock*Noise, Lock*Site, Lock*Human | 13 | 185.94 | -345.18 | 0.21 | 0.14 |
|  | Lock, Noise, Site, Human, Temperature, Lock*Noise, Lock*Site, Lock*Human | 14 | 186.93 | -345.05 | 0.35 | 0.13 |
|  | Lock, Noise, Site, Human, Count*Lock, Lock*Noise, Lock*Site, Lock*Human | 14 | 186.80 | -344.80 | 0.60 | 0.12 |
|  | Lock, Noise, Site, Human, Count*Lock, Lock*Noise, Lock*Human | 12 | 184.29 | -343.99 | 1.40 | 0.08 |
|  | Lock, Noise, Site, Human, Temperature, Count*Lock, Lock*Noise, Lock*Site, Lock*Human | 15 | 187.23 | -343.53 | 1.86 | 0.06 |
|  | Lock, Noise, Site, Human, Temperature, Count*Lock, Lock*Noise, Lock*Human | 13 | 185.03 | -343.37 | 2.02 | 0.06 |
|  | Lock, Noise, Site, Human, Count*Lock, Lock*Site, Lock*Human | 13 | 184.63 | -342.56 | 2.84 | 0.04 |
|  | Lock, Noise, Site, Human, Lock*Site, Lock*Human | 12 | 183.15 | -341.71 | 3.69 | 0.03 |
|  | Lock, Noise, Site, Human, Temperature, Lock*Site, Lock*Human | 13 | 184.07 | -341.45 | 3.94 | 0.02 |
| **Rose-ringed**  **parakeet** | **Activity (Number of syllables/day)** ~ |  |  |  |  |  |
|  | Lock, Noise, Site, Human, Temperature, Count*Lock, Lock*Noise, Lock*Site, Lock*Human | 14 | -27201.3 | 54431.34 | 0.00 | 1 |
|  | Lock, Noise, Site, Human, Count*Lock, Lock*Noise, Lock*Site, Lock*Human | 13 | -27235.3 | 54497.28 | 65.94 | 0 |
| **Rose-ringed**  **parakeet** | **Activity Variability (CV of the number of syllables/day) ~** |  |  |  |  |  |
|  | Lock, Noise, Site, Human, Temperature, Count*Lock, Lock*Site, Lock*Human | 14 | 339.72 | -650.62 | 0.00 | 0.41 |
|  | Lock, Noise, Site, Human, Count*Lock, Lock*Site, Lock*Human | 13 | 338.39 | -650.06 | 0.56 | 0.31 |
|  | Lock, Noise, Site, Human, Temperature, Count*Lock, Lock*Noise, Lock*Site, Lock*Human | 15 | 339.87 | -648.81 | 1.81 | 0.17 |
|  | Lock, Noise, Site, Human, Count*Lock, Lock*Noise, Lock*Site, Lock*Human | 14 | 338.46 | -648.11 | 2.51 | 0.12 |
| **Graceful prinia** | **Activity (Number of syllables/day)** ~ |  |  |  |  |  |
|  | Lock, Noise, Site, Human, Temperature, Count*Lock, Lock*Noise, Lock*Site, Lock*Human | 14 | -527547.1 | 1055123 | 0.00 | 1 |
|  | Lock, Noise, Site, Human, Temperature, Count*Lock, Lock*Site, Lock*Human | 13 | -527582.4 | 1055191 | 68.52 | 0 |
| **Graceful prinia** | **Activity Variability (CV of the number of syllables/day) ~** |  |  |  |  |  |
|  | Lock, Noise, Site, Temperature | 9 | -217.52 | 453.49 | 0.00 | 0.09 |
|  | Lock, Noise, Site, Temperature, Count*Lock, Lock*Site | 12 | -214.43 | 453.64 | 0.15 | 0.08 |
|  | Lock, Noise, Site, Temperature, Count*Lock | 10 | -216.62 | 453.80 | 0.31 | 0.08 |
|  | Lock, Noise, Site, Temperature, Lock*Site | 11 | -215.60 | 453.87 | 0.37 | 0.08 |
|  | Lock, Site, Temperature, Count*Lock, Lock*Site | 11 | -215.69 | 454.05 | 0.56 | 0.07 |
|  | Lock, Site, Temperature, Count*Lock | 9 | -217.91 | 454.26 | 0.77 | 0.06 |
|  | Lock, Site, Temperature | 8 | -219.15 | 454.67 | 1.17 | 0.05 |
|  | Lock, Site, Temperature, Lock*Site | 10 | -217.23 | 455.01 | 1.51 | 0.04 |
|  | Lock, Noise, Site, Temperature, Count*Lock, Lock*Noise, Lock*Site | 13 | -214.11 | 455.14 | 1.65 | 0.04 |
|  | Lock, Noise, Site, Human, Temperature, Count*Lock, Lock*Site | 13 | -214.26 | 455.44 | 1.95 | 0.03 |
|  | Lock, Noise, Site, Human, Temperature | 10 | -217.51 | 455.58 | 2.08 | 0.03 |
|  | Lock, Noise, Site, Temperature, Lock*Noise | 10 | -217.52 | 455.59 | 2.09 | 0.03 |
|  | Lock, Site, Human, Temperature, Count*Lock, Lock*Site | 12 | -215.46 | 455.70 | 2.21 | 0.03 |
|  | Lock, Noise, Site, Temperature, Lock*Noise, Lock*Site | 12 | -215.47 | 455.72 | 2.22 | 0.03 |
|  | Noise, Site, Temperature | 8 | -219.71 | 455.78 | 2.28 | 0.03 |
|  | Lock, Noise, Site, Human, Temperature, Lock*Site | 12 | -215.50 | 455.78 | 2.28 | 0.03 |
|  | Lock, Noise, Site, Human, Temperature, Count*Lock | 11 | -216.60 | 455.86 | 2.37 | 0.03 |
|  | Lock, Noise, Site, Temperature, Count*Lock, Lock*Noise | 11 | -216.60 | 455.87 | 2.38 | 0.03 |
|  | Lock, Site, Human, Temperature, Count*Lock | 10 | -217.86 | 456.28 | 2.78 | 0.02 |
|  | Lock, Site, Human, Temperature | 9 | -219.13 | 456.72 | 3.22 | 0.02 |
|  | Lock, Site, Human, Lock*Site | 11 | -217.07 | 456.80 | 3.30 | 0.02 |
|  | Lock, Noise, Site, Human, Temperature, Count*Lock, Lock*Noise, Lock*Site | 14 | -213.88 | 456.82 | 3.33 | 0.02 |
|  | Site | 6 | -222.37 | 456.95 | 3.46 | 0.02 |
|  | Lock, Noise, Site, Human, Temperature, Count*Lock, Lock*Site, Lock*Human | 14 | -214.02 | 457.11 | 3.62 | 0.01 |
|  | Lock, Site, Human, Temperature, Count*Lock, Lock*Site, Lock*Human | 13 | -215.21 | 457.33 | 3.84 | 0.01 |
|  | Noise, Site | 7 | -221.53 | 457.35 | 3.85 | 0.01 |

Bird: Bird species. Lock: Lockdown status. Site: Site category. Human: Human activity. Count: Count down. AICc: Akaike’s information criterion corrected for small sample size. ΔAICc: the difference between the alternative model and best-fitting model. Models are ranked based on the AICc values from the best to the worst model. *: Interaction effect.

**Supplementary file 1i. Effects of predictor variables on birds’ activity based on generalized and general linear mixed models (GLMM and LMM). Estimates were calculated in % per day for the following units: Temperature – per degree, Noise – per dB, Human activity – per 1 talking event, Lockdown related parameter – per existence of the lockdown (yes/no).**

| Species | Dependent  variable | Predictors | Estimate | *z* | *p* | 95% CI | Percent |
| --- | --- | --- | --- | --- | --- | --- | --- |
| All  species | **^#^Activity-**  Number of  syllables/day | (Intercept) | 6.706 | 18.210 | < 0.001 | - | - |
|  |  | Bird_species | 0.131 | **58.938** | **< 0.001** | - | 13.997 |
|  |  | Lockdown_status | 0.270 | **2.048** | **0.041** | - | 30.996 |
|  |  | Human_activity | 0.0001 | **15.114** | **< 0.001** | - | 0.010 |
|  |  | Noise | -0.088 | **-79.861** | **< 0.001** | - | 8.424 |
|  |  | Temperature | 0.187 | **18.949** | **< 0.001** | - | 20.768 |
|  |  | Site_category_residence | -0.946 | **-2.734** | **0.006** | - | 62.394 |
|  |  | Site_category_road | -1.155 | **-3.439** | **0.001** | - | 70.549 |
|  |  | Lockdown_status*Count_down | -0.016 | **-17.871** | **< 0.001** | - | 1.651 |
|  |  | Lockdown_status*Site_category_residence | -0.152 | **-22.224** | **< 0.001** | - | 14.806 |
|  |  | Lockdown_status*Site_category_road | -0.366 | **-42.070** | **< 0.001** | - | 32.489 |
|  |  | Lockdown_status*Noise | -0.009 | **-8.416** | **< 0.001** | - | 0.959 |
|  |  | Lockdown_status*Human_activity | 0.0002 | **24.963** | **< 0.001** | - | 0.022 |
|  |  | Bird_species*Lockdown_status | 0.386 | 146.072 | **< 0.001** | - | 51.348 |
|  | **^#^Activity**  **Variability-**  CV of the  number of  syllables/day | (Intercept) | -0.808 | 4.625 | < 0.001 | -1.164, -0.418 | - |
|  |  | Bird_species | 0.212 | 15.553 | **< 0.001** | **0.185, 0.239** | - |
|  |  | Lockdown_status | 0.579 | 4.014 | **< 0.001** | **0.223, 0.892** | - |
|  |  | Noise | 0.025 | 7.884 | **< 0.001** | **0.018, 0.031** | - |
|  |  | Site_category_residence | 0.183 | 2.520 | **0.012** | **0.036, 0.325** | - |
|  |  | Site_category_road | 0.247 | 3.492 | **< 0.001** | **0.107, 0.387** | - |
|  |  | Human_activity | -0.00003 | 0.658 | 0.510 | -0.0001, 0.00005 | - |
|  |  | Temperature | -0.011 | 3.313 | **0.001** | **-0.017, -0.004** | - |
|  |  | Bird_species*Lockdown_status | -0.061 | 3.768 | **< 0.001** | **-0.093, -0.029** | - |
|  |  | Lockdown_status*Noise | -0.008 | 3.030 | **0.002** | **-0.014, -0.003** | - |
|  |  | Lockdown_status*Human_activity | -0.00007 | 1.657 | 0.097 | -0.0002, 0.00001 | - |
|  |  | Count_down*Lockdown_status | -0.001 | 1.187 | 0.235 | -0.003, 0.0007 | - |
|  |  | Lockdown_status*Site_category_residence | 0.026 | 0.633 | 0.527 | -0.051, 0.113 | - |
| Hooded crow | **Activity-**  Number of  syllables/day | (Intercept) | 9.746 | 22.573 | < 0.001 | 8.900, 10.592 | - |
|  |  | Lockdown_status | -2.293 | 11.906 | **< 0.001** | **-2.670, -1.916** | 89.904 |
|  |  | Noise | -0.062 | 32.300 | **< 0.001** | **-0.066, -0.058** | 6.012 |
|  |  | Site_category_residence | -1.057 | 2.774 | **0.006** | **-1.803, -0.310** | 65.250 |
|  |  | Site_category_road | -0.726 | 1.963 | 0.050 | -1.451, -0.001 | 51.616 |
|  |  | Human_activity | 0.00007 | 5.338 | **< 0.001** | **0.00004, 0.0001** | 0.007 |
|  |  | Count_down*Lockdown_status | 0.055 | 34.878 | **< 0.001** | **0.052, 0.058** | 5.767 |
|  |  | Lockdown_status*Noise | 0.022 | 12.732 | **< 0.001** | **0.0185, 0.025** | 2.291 |
|  |  | Lockdown_status*Site_category_residence | 0.067 | 4.873 | **< 0.001** | **0.040, 0.094** | 7.207 |
|  |  | Lockdown_status*Site_category_road | 0.235 | 16.330 | **< 0.001** | **0.207, 0.263** | 27.815 |
|  |  | Lockdown_status*Human_activity | 0.00018 | 11.500 | **< 0.001** | **0.0001, 0.0002** | 0.019 |
|  |  | Temperature | 0.013 | 0.700 | 0.484 | -0.023, 0.048 | 1.400 |
|  | **^#^Activity**  **Variability-**  CV of the  number of  syllables/day | (Intercept) | -1.248 | 4.511 | < 0.001 | -1.778, -0.688 | - |
|  |  | Lockdown_status | 0.748 | 2.591 | **0.010** | **0.161, 1.325** | - |
|  |  | Noise | 0.033 | 6.152 | **< 0.001** | **0.022, 0.043** | - |
|  |  | Site_category_residence | 0.282 | 2.726 | **0.006** | **0.076, 0.484** | - |
|  |  | Site_category_road | 0.245 | 2.456 | **0.014** | **0.049, 0.440** | - |
|  |  | Human_activity | 0.00006 | 1.370 | 0.171 | -0.00003, 0.0002 | - |
|  |  | Temperature | -0.005 | 1.307 | 0.191 | -0.012, 0.003 | - |
|  |  | Lockdown_status*Noise | -0.013 | 2.634 | **0.008** | **-0.022, -0.003** | - |
|  |  | Lockdown_status*Human_activity | -0.00017 | 3.209 | **0.001** | **-0.0003, -0.00007** | - |
|  |  | Lockdown_status*Site_category_residence | 0.059 | 1.143 | 0.253 | -0.041, 0.166 | - |
|  |  | Lockdown_status*Site_category_road | -0.060 | 1.184 | 0.237 | -0.160, 0.041 | - |
|  |  | Count_down*Lockdown_status | -0.001 | 0.907 | 0.364 | -0.003, 0.001 | - |
| Rose-ringed parakeet | **Activity-**  Number of  syllables/day | (Intercept) | 6.241 | 11.779 | < 0.001 | - | - |
|  |  | Lockdown_status | 0.831 | 4.833 | **< 0.001** | - | 129.561 |
|  |  | Human_activity | -0.0004 | -25.045 | **< 0.001** | - | 0.040 |
|  |  | Noise | -0.044 | -21.666 | **< 0.001** | - | 4.305 |
|  |  | Temperature | 0.124 | 8.265 | **< 0.001** | - | 13.202 |
|  |  | Site_category_residence | -0.519 | -1.066 | 0.287 | - | 40.893 |
|  |  | Site_category_road | -1.614 | -3.415 | **0.001** | - | 81.693 |
|  |  | Lockdown_status*Count_down | -0.026 | -13.760 | **< 0.001** | - | 2.643 |
|  |  | Lockdown_status*Site_category_residence | 0.441 | 28.845 | **< 0.001** | - | 57.643 |
|  |  | Lockdown_status*Site_category_road | 0.571 | 36.727 | **< 0.001** | - | 80.854 |
|  |  | Lockdown_status*Noise | -0.026 | -14.157 | **< 0.001** | - | 2.720 |
|  |  | Lockdown_status*Human_activity | 0.001 | 53.144 | **< 0.001** | - | 0.107 |
|  | **^#^Activity**  **Variability-**  CV of the  number of  syllables/day | (Intercept) | -0.353 | 2.031 | 0.042 | -0.695, -0.009 | - |
|  |  | Lockdown_status | 0.287 | 2.797 | **0.005** | **0.079, 0.506** | - |
|  |  | Noise | 0.019 | 6.739 | **< 0.001** | **0.014, 0.026** | - |
|  |  | Site_category_residence | 0.117 | 1.219 | 0.223 | -0.073, 0.304 | - |
|  |  | Site_category_road | 0.256 | 2.759 | **0.006** | **0.070, 0.437** | - |
|  |  | Human_activity | 0.000 | 1.103 | 0.270 | -0.00004, 0.0001 | - |
|  |  | Temperature | -0.006 | 1.657 | 0.098 | -0.014, 0.001 | - |
|  |  | Count_down*Lockdown_status | -0.004 | 4.140 | **< 0.001** | **-0.006, -0.002** | - |
|  |  | Lockdown_status*Site_category_residence | -0.052 | 1.499 | 0.134 | -0.121, 0.017 | - |
|  |  | Lockdown_status*Site_category_road | -0.121 | 3.470 | **0.001** | **-0.189, -0.052** | - |
|  |  | Lockdown_status*Human_activity | 0.000 | 3.596 | **< 0.001** | **-0.0002, -0.00006** | - |
|  |  | Lockdown_status*Noise | -0.002 | 0.507 | 0.612 | -0.009, 0.005 | - |
| Graceful prinia | **Activity-**  Number of  syllables/day | (Intercept) | 6.972 | 18.936 | < 0.001 | - | - |
|  |  | Lockdown_status | 1.123 | 8.513 | **< 0.001** | - | 207.406 |
|  |  | Human_activity | 0.0001 | 15.114 | **< 0.001** | - | 0.010 |
|  |  | Noise | -0.088 | -79.861 | **< 0.001** | - | 8.424 |
|  |  | Temperature | 0.187 | 18.949 | **< 0.001** | - | 20.563 |
|  |  | Site_category_residence | -0.946 | -2.734 | **0.006** | - | 61.783 |
|  |  | Site_category_road | -1.155 | -3.439 | **0.001** | - | 69.864 |
|  |  | Lockdown_status*Count_down | -0.016 | -17.871 | **< 0.001** | - | 1.635 |
|  |  | Lockdown_status*Site_category_residence | -0.152 | -22.224 | **< 0.001** | - | 14.665 |
|  |  | Lockdown_status*Site_category_road | -0.366 | -42.070 | **< 0.001** | - | 32.182 |
|  |  | Lockdown_status*Noise | -0.009 | -8.416 | **< 0.001** | - | 0.950 |
|  |  | Lockdown_status*Human_activity | 0.0002 | 24.963 | **< 0.001** | - | 0.021 |
|  | **^#^Activity**  **Variability-**  CV of the  number of  syllables/day | (Intercept) | 1.172 | 2.048 | 0.041 | 0.0127, 2.247 | - |
|  |  | Lockdown_status | -0.190 | 0.677 | 0.498 | -0.751, 0.407 | - |
|  |  | Noise | 0.015 | 1.582 | 0.114 | -0.004, 0.035 | - |
|  |  | Site_category_residence | 0.191 | 0.930 | 0.353 | -0.210, 0.594 | - |
|  |  | Site_category_road | 0.531 | 2.495 | **0.013** | **0.117, 0.953** | - |
|  |  | Temperature | -0.029 | 2.528 | **0.012** | **-0.051, -0.006** | - |
|  |  | Count_down*Lockdown_status | 0.005 | 1.544 | 0.123 | -0.002, 0.011 | - |
|  |  | Lockdown_status*Site_category_residence | 0.147 | 1.278 | 0.201 | -0.079, 0.374 | - |
|  |  | Lockdown_status*Site_category_road | 0.260 | 2.044 | **0.041** | **0.011, 0.512** | - |
|  |  | Lockdown_status*Noise | -0.006 | 0.487 | 0.627 | -0.029, 0.018 | - |
|  |  | Human_activity | -0.00005 | 0.464 | 0.642 | -0.0003, 0.0002 | - |
|  |  | Lockdown_status*Human_activity | 0.0001 | 0.698 | 0.485 | -0.0003, 0.0004 | - |

CV: coefficient of variance. *: Interaction effect. 95% confidence intervals of the parameters that did not overlap zero are indicated in bold. **#**: model average.

**Supplementary file 1j.** **Results from post hoc tests comparing the differences in activity and activity variability between no lockdown period and lockdown period in all species for each site. Estimates were calculated in % per day for the following units: Temp – per degree, Noise – per dB, Human activity – per 1 Human_activitying event, Lockdown related parameter – per existence of the lockdown (yes/no).**

| Species | Dependent variable | Site category | Estimate | *P* | Percent |
| --- | --- | --- | --- | --- | --- |
| All species | **Activity**-  Number of syllables/day | Road | -0.073 | 0.767 | 7.040 |
|  |  | Residence | -0.286 | 0.244 | 24.874 |
|  |  | Park | -0.438 | 0.074 | 35.467 |
|  | **Activity variability**-  CV of the number of syllables/day | Road | -0.037 | 0.260 | - |
|  |  | Residence | -0.016 | 0.663 | - |
|  |  | Park | -0.0006 | 0.988 | - |
| Hooded crow | **Activity**-  Number of syllables/day | Road | 0.155 | 0.659 | 16.766 |
|  |  | Residence | 0.323 | 0.359 | 38.127 |
|  |  | Park | 0.390 | 0.268 | 47.698 |
|  | **Activity variability**-  CV of the number of syllables/day | Road | -0.046 | 0.217 | - |
|  |  | Residence | -0.078 | 0.080 | - |
|  |  | Park | -0.034 | 0.461 | - |
| Rose-ringed parakeet | **Activity**-  Number of events/day | Road | -0.209 | 0.138 | 18.860 |
|  |  | Residence | -0.079 | 0.577 | 7.596 |
|  |  | Park | 0.362 | **0.010** | 43.620 |
|  | **Activity variability**-  CV of the number of syllables/day | Road | -0.029 | 0.332 | - |
|  |  | Residence | -0.070 | **0.038** | - |
|  |  | Park | -0.128 | **0.0003** | - |
| Graceful prinia | **Activity-**  Number of syllables/day | Road | -0.464 | 0.302 | 37.124 |
|  |  | Residence | -1.838 | **<0.0001** | 84.086 |
|  |  | Park | -1.922 | **<0.0001** | 85.369 |
|  | **Activity variability**-  CV of the number of syllables/day | Road | -0.038 | 0.781 | - |
|  |  | Residence | 0.142 | 0.285 | - |
|  |  | Park | 0.303 | **0.030** | - |

CV: coefficient of variance

**Supplementary file 1k. The sampling time and daily number of syllables including crow's vocalizations for each site.**

| Time | Site3 | Site4 | Site5 | Site6 | Site7 | Site8 | Site9 | Site10 | Site11 | Site12 | Site13 | Site14 | Site15 | Site16 | Site17 | Site18 | Site19 |
| --- | --- | --- | --- | --- | --- | --- | --- | --- | --- | --- | --- | --- | --- | --- | --- | --- | --- |
| 2020/3/25 | 130 | 20 | 40 | 106 | 36 | 230 | 142 | 144 | - | - | - | - | - | - | - | - | - |
| 2020/3/26 | 34 | 26 | 44 | 50 | 54 | 212 | 164 | 178 | - | - | - | - | - | - | - | - | - |
| 2020/3/27 | 18 | 14 | 38 | 60 | 36 | 264 | 180 | 190 | - | - | - | - | - | - | - | - | - |
| 2020/3/28 | 42 | 24 | 28 | 96 | 20 | 348 | 218 | 200 | - | - | - | - | - | - | - | - | - |
| 2020/3/30 | 26 | 10 | 32 | 28 | 38 | 306 | 154 | 114 | - | - | - | - | - | - | - | - | - |
| 2020/3/31 | 38 | 10 | 4 | 34 | 38 | 306 | 142 | 96 | 82 | 16 | 140 | 46 | 84 | 34 | 58 | 118 | 170 |
| 2020/4/1 | 36 | 16 | 10 | 34 | 36 | 190 | 160 | 58 | 72 | 18 | 150 | 44 | 82 | 30 | 64 | 142 | 198 |
| 2020/4/2 | 40 | 8 | 8 | 36 | 34 | 204 | 138 | 52 | 80 | 28 | 254 | 32 | 82 | 46 | 74 | 94 | 154 |
| 2020/4/3 | 18 | 16 | 16 | 64 | 26 | 238 | 168 | 152 | 74 | 64 | 302 | 30 | 88 | 58 | 111 | 162 | 230 |
| 2020/4/4 | 72 | 10 | 12 | 142 | 58 | 230 | 168 | 188 | 174 | 86 | 258 | 72 | 90 | 94 | 176 | 170 | 182 |
| 2020/4/5 | 40 | 12 | 14 | 42 | 30 | 166 | 120 | 82 | 98 | 32 | 106 | 32 | 54 | 32 | 88 | 52 | 162 |
| 2020/4/6 | 46 | 6 | 26 | 22 | 30 | 200 | 132 | 80 | 106 | 22 | 94 | 36 | 62 | 30 | 62 | 104 | 154 |
| 2020/4/7 | 62 | 8 | 30 | 48 | 36 | 180 | 160 | 90 | 84 | 24 | 104 | 40 | 36 | 32 | 44 | 78 | 146 |
| 2020/4/8 | 116 | 12 | 40 | 82 | 40 | 156 | 188 | - | 158 | 30 | 138 | 64 | 70 | 56 | 92 | 166 | 210 |
| 2020/4/9 | - | - | - | - | - | - | - | - | - | 44 | 110 | - | - | - | - | - | - |
| 2020/4/24 | - | 32 | 24 | 50 | - | 160 | 122 | 68 | 68 | 34 | - | - | - | 46 | - | - | - |
| 2020/4/25 | - | 38 | 32 | 126 | - | 210 | 160 | 124 | 80 | 42 | - | - | - | 72 | - | - | - |
| 2020/4/26 | - | 18 | 2 | 46 | - | 214 | 307 | 58 | 82 | 16 | - | - | - | 26 | - | - | - |
| 2020/4/27 | - | 44 | 8 | 44 | - | 198 | 200 | 112 | 80 | 18 | - | - | - | 24 | - | - | - |
| 2020/4/28 | - | 28 | 12 | 42 | - | 150 | 234 | 108 | 82 | 40 | - | - | - | 26 | - | - | - |
| 2020/4/29 | - | 48 | 24 | 104 | - | 208 | 510 | 242 | 186 | 100 | - | - | - | 68 | - | - | - |
| 2020/4/30 | - | 26 | 20 | 52 | - | 144 | 338 | 138 | 90 | 28 | - | - | - | 26 | - | - | - |
| 2020/5/1 | - | 48 | 44 | 62 | - | 196 | 278 | 252 | 114 | 22 | - | - | - | 18 | - | - | - |
| 2020/5/2 | - | 66 | 30 | 146 | - | 192 | 210 | 206 | 156 | 104 | - | - | - | 46 | - | - | - |
| 2020/5/3 | - | - | 20 | 94 | - | 134 | 146 | - | - | - | - | - | - | 20 | - | - | - |
| 2020/5/7 | - | 92 | 20 | 86 | - | 98 | 170 | 102 | 88 | 26 | - | - | - | 16 | - | 62 | - |
| 2020/5/8 | - | 86 | 26 | 112 | - | 130 | 192 | 102 | 134 | 28 | - | - | - | 18 | - | 74 | - |
| 2020/5/9 | - | 84 | 20 | 120 | - | 112 | 256 | 94 | 190 | 18 | - | - | - | 14 | - | 96 | - |
| 2020/5/10 | - | 60 | 20 | 88 | - | 104 | 98 | 66 | 162 | 20 | - | - | - | 22 | - | 44 | - |
| 2020/5/11 | - | 64 | 10 | 76 | - | 104 | 156 | 84 | 150 | 20 | - | - | - | 36 | - | 56 | - |
| 2020/5/12 | - | 22 | 16 | 102 | - | 106 | 390 | 78 | 142 | 10 | - | - | - | 30 | - | 84 | - |
| 2020/5/13 | - | 42 | 16 | 64 | - | 140 | 310 | 80 | 128 | 16 | - | - | - | 10 | - | 46 | - |
| 2020/5/14 | - | 72 | 10 | 82 | - | 144 | 376 | 82 | 174 | 30 | - | - | - | 14 | - | 48 | - |
| 2020/5/15 | - | 44 | 22 | 108 | - | 194 | 252 | 86 | 214 | 40 | - | - | - | 28 | - | 48 | - |
| 2020/5/16 | - | 40 | 10 | 130 | - | 144 | 238 | 76 | 136 | 70 | - | - | - | 38 | - | 62 | - |
| 2020/5/21 | 144 | 38 | 26 | 214 | 70 | 200 | 118 | 88 | 212 | 30 | 108 | 42 | 250 | 68 | 56 | 36 | 98 |
| 2020/5/22 | 150 | 60 | 54 | 222 | 70 | 186 | 168 | 120 | 198 | 36 | 160 | 42 | 268 | 38 | 110 | 32 | 152 |
| 2020/5/23 | 174 | 54 | 24 | 208 | 154 | 198 | 152 | 148 | 261 | 54 | 146 | 142 | 278 | 72 | 110 | 78 | 162 |
| 2020/5/24 | 96 | 44 | 38 | 114 | 106 | 144 | 186 | 78 | 164 | 22 | 100 | 24 | 238 | 40 | 74 | 32 | 164 |
| 2020/5/25 | 100 | 42 | 52 | 108 | 46 | 158 | 156 | 98 | 202 | 28 | 138 | 58 | 172 | 64 | 132 | 42 | 152 |
| 2020/5/26 | 78 | 68 | 44 | 104 | 62 | 176 | 134 | 74 | 194 | 44 | 116 | 60 | 172 | 34 | 118 | 42 | 134 |
| 2020/5/27 | 84 | 108 | 30 | 114 | 38 | 216 | 112 | 86 | 156 | 46 | 134 | 36 | 224 | 14 | 86 | 50 | 102 |
| 2020/5/28 | 102 | 30 | 52 | 134 | 48 | 212 | 124 | 106 | 160 | 72 | 168 | 62 | 176 | 34 | 124 | 34 | 186 |
| 2020/5/29 | 120 | 58 | 38 | - | 60 | 280 | 146 | 117 | - | 84 | 174 | 102 | 218 | 78 | 152 | 86 | 252 |
| 2020/5/30 | - | 36 | - | - | - | - | - | - | - | - | 144 | 86 | 188 | 74 | 130 | 58 | 242 |

**Supplementary file 1l. The sampling time and daily number of syllables including parakeet's vocalizations for each site.**

| Time | Site3 | Site4 | Site5 | Site6 | Site7 | Site8 | Site9 | Site10 | Site11 | Site12 | Site13 | Site14 | Site15 | Site16 | Site17 | Site18 | Site19 |
| --- | --- | --- | --- | --- | --- | --- | --- | --- | --- | --- | --- | --- | --- | --- | --- | --- | --- |
| 2020/3/25 | 76 | 164 | 726 | 88 | 110 | 1168 | 44 | 556 | - | - | - | - | - | - | - | - | - |
| 2020/3/26 | 40 | 208 | 410 | 142 | 112 | 1548 | 86 | 724 | - | - | - | - | - | - | - | - | - |
| 2020/3/27 | 62 | 224 | 340 | 270 | 100 | 3324 | 22 | 386 | - | - | - | - | - | - | - | - | - |
| 2020/3/28 | 274 | 260 | 120 | 244 | 158 | 4652 | 28 | 354 | - | - | - | - | - | - | - | - | - |
| 2020/3/30 | 50 | 188 | 316 | 138 | 52 | 2364 | 60 | 256 | - | - | - | - | - | - | - | - | - |
| 2020/3/31 | 68 | 340 | 312 | 84 | 32 | 1744 | 38 | 106 | 1610 | 110 | 724 | 194 | 194 | 4 | 38 | 16 | 434 |
| 2020/4/1 | 80 | 276 | 178 | 132 | 70 | 1898 | 20 | 122 | 1316 | 60 | 640 | 224 | 208 | 0 | 4 | 24 | 278 |
| 2020/4/2 | 72 | 442 | 268 | 152 | 42 | 2632 | 66 | 174 | 1796 | 72 | 622 | 122 | 230 | 2 | 40 | 122 | 366 |
| 2020/4/3 | 94 | 358 | 140 | 320 | 262 | 2018 | 74 | 286 | 1186 | 146 | 548 | 254 | 226 | 0 | 80 | 134 | 864 |
| 2020/4/4 | 184 | 424 | 174 | 354 | 400 | 1272 | 56 | 214 | 1728 | 136 | 446 | 214 | 164 | 238 | 224 | 142 | 1512 |
| 2020/4/5 | 64 | 408 | 104 | 336 | 28 | 1130 | 52 | 326 | 1088 | 18 | 536 | 160 | 218 | 2 | 44 | 48 | 478 |
| 2020/4/6 | 46 | 392 | 118 | 138 | 54 | 1816 | 64 | 42 | 1890 | 80 | 488 | 158 | 614 | 0 | 114 | 50 | 464 |
| 2020/4/7 | 108 | 482 | 218 | 206 | 40 | 1052 | 12 | 98 | 1458 | 42 | 392 | 180 | 356 | 0 | 90 | 14 | 790 |
| 2020/4/8 | 112 | 316 | 124 | 316 | 38 | 1570 | 24 | - | 3020 | 36 | 646 | 248 | 288 | 12 | 40 | 38 | 1360 |
| 2020/4/9 | - | - | - | - | - | - | - | - | - | 194 | 388 | - | - | - | - | - | - |
| 2020/4/24 | - | 204 | 128 | 212 | - | 1776 | 86 | 764 | 1696 | 64 | - | - | - | 20 | - | - | - |
| 2020/4/25 | - | 302 | 136 | 364 | - | 2156 | 24 | 762 | 1286 | 60 | - | - | - | 34 | - | - | - |
| 2020/4/26 | - | 190 | 262 | 254 | - | 1980 | 10 | 620 | 1678 | 14 | - | - | - | 0 | - | - | - |
| 2020/4/27 | - | 132 | 392 | 548 | - | 2278 | 76 | 510 | 1346 | 2 | - | - | - | 22 | - | - | - |
| 2020/4/28 | - | 176 | 306 | 320 | - | 2602 | 206 | 758 | 1648 | 68 | - | - | - | 8 | - | - | - |
| 2020/4/29 | - | 206 | 324 | 384 | - | 2562 | 140 | 1724 | 2494 | 238 | - | - | - | 44 | - | - | - |
| 2020/4/30 | - | 148 | 158 | 218 | - | 2480 | 14 | 826 | 2310 | 2 | - | - | - | 16 | - | - | - |
| 2020/5/1 | - | 242 | 96 | 780 | - | 2186 | 128 | 918 | 1682 | 144 | - | - | - | 62 | - | - | - |
| 2020/5/2 | - | 392 | 152 | 462 | - | 1476 | 70 | 1050 | 2384 | 124 | - | - | - | 38 | - | - | - |
| 2020/5/3 | - | - | 358 | 202 | - | 2108 | 142 | - | - | - | - | - | - | 0 | - | - | - |
| 2020/5/7 | - | 118 | 370 | 242 | - | 3778 | 146 | 946 | 1894 | 18 | - | - | - | 0 | - | 16 | - |
| 2020/5/8 | - | 158 | 456 | 718 | - | 2628 | 182 | 596 | 2182 | 70 | - | - | - | 18 | - | 20 | - |
| 2020/5/9 | - | 254 | 482 | 1408 | - | 2652 | 320 | 2374 | 3292 | 16 | - | - | - | 38 | - | 126 | - |
| 2020/5/10 | - | 422 | 458 | 912 | - | 3238 | 178 | 966 | 1456 | 76 | - | - | - | 14 | - | 28 | - |
| 2020/5/11 | - | 526 | 782 | 260 | - | 2090 | 238 | 1098 | 1990 | 46 | - | - | - | 42 | - | 26 | - |
| 2020/5/12 | - | 400 | 464 | 488 | - | 3064 | 138 | 126 | 2410 | 20 | - | - | - | 16 | - | 74 | - |
| 2020/5/13 | - | 236 | 758 | 490 | - | 2556 | 164 | 552 | 3098 | 116 | - | - | - | 6 | - | 46 | - |
| 2020/5/14 | - | 312 | 338 | 410 | - | 3016 | 106 | 548 | 2960 | 62 | - | - | - | 46 | - | 184 | - |
| 2020/5/15 | - | 564 | 1158 | 616 | - | 2636 | 152 | 954 | 4024 | 114 | - | - | - | 4 | - | 108 | - |
| 2020/5/16 | - | 438 | 308 | 242 | - | 2940 | 90 | 690 | 2584 | 150 | - | - | - | 74 | - | 814 | - |
| 2020/5/21 | 330 | 282 | 508 | 260 | 174 | 2492 | 118 | 654 | 3558 | 88 | 334 | 188 | 688 | 0 | 18 | 74 | 244 |
| 2020/5/22 | 738 | 478 | 1198 | 656 | 66 | 2904 | 358 | 650 | 3162 | 218 | 586 | 96 | 1224 | 12 | 26 | 212 | 600 |
| 2020/5/23 | 302 | 370 | 1156 | 968 | 188 | 2460 | 104 | 786 | 4298 | 296 | 484 | 326 | 902 | 56 | 248 | 344 | 670 |
| 2020/5/24 | 184 | 216 | 508 | 360 | 208 | 1846 | 30 | 354 | 1906 | 38 | 144 | 188 | 438 | 46 | 54 | 50 | 460 |
| 2020/5/25 | 666 | 324 | 810 | 416 | 134 | 2650 | 88 | 698 | 3178 | 112 | 360 | 122 | 654 | 32 | 50 | 94 | 730 |
| 2020/5/26 | 314 | 492 | 1240 | 484 | 124 | 2530 | 210 | 540 | 4200 | 68 | 260 | 278 | 1238 | 32 | 60 | 82 | 722 |
| 2020/5/27 | 364 | 282 | 1968 | 354 | 148 | 1992 | 222 | 198 | 4018 | 98 | 480 | 126 | 534 | 84 | 60 | 16 | 492 |
| 2020/5/28 | 374 | 226 | 1348 | 370 | 288 | 2056 | 188 | 474 | 4046 | 98 | 446 | 98 | 528 | 0 | 72 | 80 | 646 |
| 2020/5/29 | 570 | 448 | 416 | - | 534 | 1380 | 214 | 612 | - | 400 | 786 | 772 | 430 | 132 | 104 | 100 | 724 |
| 2020/5/30 | - | 480 | - | - | - | - | - | - | - | - | 818 | 420 | 694 | 272 | 22 | 76 | 630 |

**Supplementary file 1m. The sampling time and daily number of syllables including prinia's vocalizations for each site.**

| Time | Site3 | Site4 | Site5 | Site6 | Site7 | Site8 | Site9 | Site10 | Site11 | Site12 | Site13 | Site14 | Site15 | Site16 | Site17 | Site18 | Site19 |
| --- | --- | --- | --- | --- | --- | --- | --- | --- | --- | --- | --- | --- | --- | --- | --- | --- | --- |
| 2020/3/25 | 142 | 356 | 416 | 892 | 32 | 428 | 2746 | 4650 | - | - | - | - | - | - | - | - | - |
| 2020/3/26 | 222 | 384 | 800 | 2454 | 0 | 1086 | 4038 | 3466 | - | - | - | - | - | - | - | - | - |
| 2020/3/27 | 0 | 202 | 234 | 1002 | 30 | 1228 | 4710 | 2904 | - | - | - | - | - | - | - | - | - |
| 2020/3/28 | 380 | 288 | 674 | 870 | 124 | 718 | 2184 | 2568 | - | - | - | - | - | - | - | - | - |
| 2020/3/30 | 84 | 406 | 688 | 1174 | 0 | 204 | 4866 | 400 | - | - | - | - | - | - | - | - | - |
| 2020/3/31 | 98 | 4 | 296 | 2122 | 0 | 1256 | 4314 | 0 | 324 | 728 | 118 | 0 | 5488 | 0 | 0 | 0 | 5430 |
| 2020/4/1 | 0 | 0 | 4 | 142 | 0 | 60 | 4048 | 0 | 0 | 526 | 60 | 44 | 780 | 0 | 0 | 0 | 4460 |
| 2020/4/2 | 0 | 46 | 96 | 302 | 42 | 96 | 5356 | 16 | 264 | 1500 | 26 | 48 | 3014 | 0 | 0 | 96 | 3354 |
| 2020/4/3 | 264 | 204 | 346 | 246 | 0 | 136 | 5906 | 224 | 196 | 1746 | 72 | 202 | 2662 | 0 | 38 | 0 | 2082 |
| 2020/4/4 | 430 | 232 | 534 | 612 | 0 | 154 | 10204 | 228 | 0 | 2446 | 26 | 282 | 5378 | 16 | 0 | 42 | 6074 |
| 2020/4/5 | 504 | 174 | 378 | 492 | 0 | 166 | 6204 | 0 | 856 | 860 | 140 | 0 | 4484 | 0 | 60 | 0 | 5202 |
| 2020/4/6 | 248 | 274 | 708 | 346 | 0 | 474 | 3380 | 290 | 90 | 256 | 0 | 16 | 7018 | 0 | 400 | 0 | 2068 |
| 2020/4/7 | 1054 | 660 | 744 | 234 | 60 | 60 | 5556 | 190 | 0 | 38 | 0 | 0 | 4742 | 0 | 38 | 0 | 2414 |
| 2020/4/8 | 1094 | 386 | 198 | 294 | 34 | 12 | 3084 | - | 58 | 178 | 234 | 84 | 3268 | 0 | 94 | 0 | 3424 |
| 2020/4/9 | - | - | - | - | - | - | - | - | - | 0 | 11 | - | - | - | - | - | - |
| 2020/4/24 | - | 0 | 548 | 186 | - | 1362 | 10606 | 5304 | 246 | 38 | - | - | - | 0 | - | - | - |
| 2020/4/25 | - | 184 | 164 | 186 | - | 878 | 4620 | 3684 | 68 | 0 | - | - | - | 0 | - | - | - |
| 2020/4/26 | - | 68 | 94 | 214 | - | 694 | 3852 | 6412 | 178 | 78 | - | - | - | 0 | - | - | - |
| 2020/4/27 | - | 74 | 196 | 274 | - | 916 | 4830 | 4664 | 44 | 0 | - | - | - | 0 | - | - | - |
| 2020/4/28 | - | 0 | 316 | 1198 | - | 3260 | 3732 | 738 | 112 | 6 | - | - | - | 0 | - | - | - |
| 2020/4/29 | - | 274 | 194 | 554 | - | 2410 | 2748 | 796 | 256 | 30 | - | - | - | 0 | - | - | - |
| 2020/4/30 | - | 0 | 460 | 134 | - | 3578 | 2302 | 20 | 68 | 0 | - | - | - | 0 | - | - | - |
| 2020/5/1 | - | 286 | 352 | 346 | - | 7306 | 2722 | 1188 | 22 | 28 | - | - | - | 0 | - | - | - |
| 2020/5/2 | - | 804 | 60 | 430 | - | 8254 | 4240 | 1072 | 132 | 100 | - | - | - | 0 | - | - | - |
| 2020/5/3 | - | - | 72 | 172 | - | 8738 | 3190 | - | - | - | - | - | - | 18 | - | - | - |
| 2020/5/7 | - | 0 | 4 | 20 | - | 6534 | 1942 | 136 | 1014 | 42 | - | - | - | 0 | - | 0 | - |
| 2020/5/8 | - | 94 | 20 | 266 | - | 6740 | 3854 | 50 | 716 | 240 | - | - | - | 0 | - | 26 | - |
| 2020/5/9 | - | 0 | 0 | 40 | - | 5006 | 3246 | 0 | 728 | 152 | - | - | - | 14 | - | 0 | - |
| 2020/5/10 | - | 0 | 12 | 0 | - | 15374 | 1736 | 0 | 998 | 34 | - | - | - | 0 | - | 0 | - |
| 2020/5/11 | - | 0 | 12 | 44 | - | 15458 | 420 | 178 | 2282 | 4 | - | - | - | 0 | - | 0 | - |
| 2020/5/12 | - | 22 | 66 | 0 | - | 11372 | 1072 | 0 | 1576 | 52 | - | - | - | 0 | - | 0 | - |
| 2020/5/13 | - | 0 | 234 | 182 | - | 9788 | 904 | 0 | 878 | 110 | - | - | - | 0 | - | 6 | - |
| 2020/5/14 | - | 0 | 1332 | 344 | - | 7180 | 1352 | 0 | 1650 | 362 | - | - | - | 0 | - | 228 | - |
| 2020/5/15 | - | 0 | 1850 | 168 | - | 5148 | 3222 | 0 | 2734 | 756 | - | - | - | 0 | - | 416 | - |
| 2020/5/16 | - | 0 | 856 | 24 | - | 3290 | 3622 | 0 | 1692 | 412 | - | - | - | 0 | - | 168 | - |
| 2020/5/21 | 0 | 588 | 0 | 462 | 784 | 5114 | 3332 | 0 | 230 | 0 | 56 | 188 | 3626 | 0 | 32 | 0 | 1686 |
| 2020/5/22 | 182 | 292 | 46 | 478 | 130 | 4210 | 2282 | 20 | 830 | 0 | 34 | 236 | 3326 | 0 | 170 | 0 | 1880 |
| 2020/5/23 | 154 | 458 | 162 | 70 | 142 | 4248 | 2958 | 0 | 1236 | 58 | 0 | 254 | 2456 | 16 | 500 | 148 | 4306 |
| 2020/5/24 | 60 | 156 | 120 | 138 | 0 | 2668 | 2746 | 0 | 892 | 10 | 52 | 142 | 840 | 0 | 48 | 0 | 1844 |
| 2020/5/25 | 54 | 444 | 10 | 1348 | 0 | 2690 | 1648 | 0 | 542 | 0 | 110 | 172 | 848 | 10 | 0 | 0 | 924 |
| 2020/5/26 | 196 | 0 | 1506 | 1216 | 316 | 2644 | 1100 | 218 | 72 | 0 | 0 | 78 | 1414 | 0 | 102 | 0 | 436 |
| 2020/5/27 | 114 | 104 | 650 | 714 | 124 | 3606 | 848 | 170 | 564 | 6 | 0 | 144 | 2684 | 0 | 22 | 0 | 244 |
| 2020/5/28 | 106 | 116 | 2098 | 1294 | 2864 | 4860 | 1380 | 0 | 1932 | 0 | 0 | 186 | 628 | 0 | 20 | 0 | 788 |
| 2020/5/29 | 0 | 280 | 596 | - | 1368 | 4470 | 1490 | 0 | - | 24 | 0 | 226 | 268 | 0 | 114 | 0 | 2210 |
| 2020/5/30 | - | 74 | - | - | - | - | - | - | - | - | 44 | 226 | 428 | 0 | 96 | 0 | 3286 |

**Supplementary file 1n. Effects of predictor variables on birds’ activity based on generalized and general linear mixed models (GLMM and LMM). Estimates were calculated in % per day for the following units: Temperature – per degree, Noise – per dB, Human activity – per 1 talking event, Lockdown related parameter – per existence of the lockdown (yes/no).**

| Species | Dependent  variable | Predictors | Estimate | *z* | *t* | *p* | 95% CI | Percent |
| --- | --- | --- | --- | --- | --- | --- | --- | --- |
| All species | **Activity-**  Number of  events/day | (Intercept) | 7.042 | 18.271 | - | < 0.001 | - | - |
|  |  | Bird_species | -0.642 | -76.465 | **-** | **< 0.001** | - | 47.38 |
|  |  | Lockdown_status | 0.209 | 1.259 | - | 0.208 | - | 23.24 |
|  |  | Human_activity | -0.0001 | -3.351 | **-** | **0.001** | **-** | 0.01 |
|  |  | Noise | -0.049 | -14.612 | **-** | **< 0.001** | - | 4.78 |
|  |  | Temperature | 0.052 | 3.975 | **-** | **< 0.001** | **-** | 5.34 |
|  |  | Site_category_residence | -0.722 | -2.739 | **-** | **0.006** | **-** | 51.42 |
|  |  | Site_category_road | -0.920 | -3.596 | **-** | **< 0.001** | **-** | 60.15 |
|  |  | Lockdown_status*Count_down | 0.008 | 3.214 | **-** | **0.001** | **-** | 0.80 |
|  |  | Lockdown_status*Site_category_residence | -0.092 | -3.565 | **-** | **< 0.001** | **-** | 8.79 |
|  |  | Lockdown_status*Site_category_road | 0.039 | 1.495 | - | 0.135 | **-** | 3.98 |
|  |  | Lockdown_status*Noise | -0.021 | -7.266 | **-** | **< 0.001** | **-** | 2.08 |
|  |  | Lockdown_status*Human_activity | 0.0004 | 14.956 | **-** | **< 0.001** | **-** | 0.04 |
|  |  | Bird_species*Lockdown_status | 0.269 | 27.540 | **-** | **< 0.001** | **-** | 30.87 |
|  | **^#^Activity**  **Variability-**  CV of the  number of  events/day | (Intercept) | -0.689 | - | -4.312 | < 0.001 | **-**1.0171, -0.2238 | - |
|  |  | Bird_species | 0.210 | - | 15.391 | **< 0.001** | **0.1815, 0.2372** | - |
|  |  | Lockdown_status | 0.378 | - | 2.898 | **0.004** | **-0.0542, 0.6611** | - |
|  |  | Human_activity | -0.0001 | - | -2.021 | **0.043** | **-0.0001, 0.00002** | - |
|  |  | Noise | 0.023 | - | 7.956 | **< 0.001** | **0.0151, 0.0288** | - |
|  |  | Temperature | -0.017 | - | -4.990 | **< 0.001** | **-0.026, -0.0096** | - |
|  |  | Site_category_residence | 0.170 | - | 2.485 | **0.023** | **0.0184, 0.3051** | - |
|  |  | Site_category_road | 0.227 | - | 3.389 | **0.003** | **0.0763, 0.3573** | - |
|  |  | Lockdown_status*Noise | -0.005 | - | -2.094 | **0.036** | **-0.0117, 0.0003** | - |
|  |  | Bird_species*Lockdown_status | -0.056 | - | -3.497 | **< 0.001** | **-0.0866, -0.0237** | - |
| Hooded  crow | #Activity-  Number of  events/day | (Intercept) | 6.446 | 19.191 | - | < 0.001 | 5.7095, 7.3195 | - |
|  |  | Lockdown_type | 0.007 | 0.033 | - | 0.974 | -0.4547, 0.4164 | 0.70 |
|  |  | Human_activity | -0.0001 | -4.264 | **-** | **< 0.001** | **-0.0002, -0.0001** | 0.01 |
|  |  | Noise | -0.027 | -6.061 | **-** | **< 0.001** | **-0.0354, -0.0178** | 2.66 |
|  |  | Site_category_residence | -0.746 | -2.247 | **-** | **0.025** | **-1.3985, -0.0938** | 52.57 |
|  |  | Site_category_road | -0.765 | -2.375 | **-** | **0.018** | **-1.3971, -0.1315** | 53.47 |
|  |  | Lockdown_status*Count_down | 0.013 | 5.209 | **-** | **< 0.001** | **0.0082, 0.0192** | 1.31 |
|  |  | Lockdown_status*Site_category_residence | -0.089 | -2.477 | **-** | **0.013** | **-0.1596, -0.0166** | 8.52 |
|  |  | Lockdown_status*Site_category_road | 0.158 | 4.556 | **-** | **< 0.001** | **0.0890, 0.2275** | 17.12 |
|  |  | Lockdown_status*Noise | -0.012 | -3.221 | **-** | **0.001** | **-0.0194, -0.0046** | 1.19 |
|  |  | Lockdown_status*Human_activity | 0.0003 | 8.887 | **-** | **< 0.001** | **0.0003, 0.0004** | 0.03 |
|  | **^#^Activity**  **Variability-**  CV of the  number of  events/day | (Intercept) | -1.181 | - | -6.232 | < 0.001 | -1.649, -0.639 | - |
|  |  | Lockdown_status | 0.694 | - | 4.741 | **< 0.001** | **0.163, 1.068** | - |
|  |  | Human_activity | 0.0000 | - | 0.721 | 0.471 | -0.0001, 0.0001 | - |
|  |  | Noise | 0.032 |  | 9.356 | **< 0.001** | **0.022, 0.039** | - |
|  |  | Temperature | -0.007 | - | -1.795 | 0.073 | -0.017, 0.002 | - |
|  |  | Site_category_residence | 0.306 | - | 3.157 | **0.006** | **0.071, 0.488** | - |
|  |  | Site_category_road | 0.235 | - | 2.497 | **0.023** | **0.038, 0.422** | - |
|  |  | Lockdown_status*Noise | -0.012 | - | -4.005 | **< 0.001** | **-0.018, -0.004** | - |
|  |  | Lockdown_status*Human_activity | -0.0001 | - | -2.378 | **0.018** | **-0.0002, -0.00001** | - |
| Rose-ringed  parakeet | **^#^Activity-**  Number of  events/day | (Intercept) | 5.058 | 9.767 | - | < 0.001 | - | - |
|  |  | Lockdown_status | 0.276 | 1.051 | - | 0.293 | - | 31.78 |
|  |  | Human_activity | -0.0003 | -6.814 | **-** | **< 0.001** | **-** | 0.03 |
|  |  | Noise | -0.050 | -8.109 | **-** | **< 0.001** | - | 4.88 |
|  |  | Temperature | 0.090 | 6.584 | **-** | **< 0.001** | **-** | 9.42 |
|  |  | Site_category_residence | -0.479 | -1.146 | - | 0.252 | - | 38.06 |
|  |  | Site_category_road | -1.172 | -2.889 | **-** | **0.004** | - | 69.03 |
|  |  | Lockdown_status*Site_category_residence | 0.390 | 8.039 | **-** | **< 0.001** | **-** | 47.70 |
|  |  | Lockdown_status*Site_category_road | 0.423 | 8.782 | **-** | **< 0.001** | **-** | 52.65 |
|  |  | Lockdown_status*Noise | -0.022 | -4.276 | **-** | **< 0.001** | **-** | 2.18 |
|  |  | Lockdown_status*Human_activity | 0.001 | 15.119 | **-** | **< 0.001** | **-** | 0.10 |
|  | **^#^Activity**  **Variability-**  CV of the  number of  events/day | (Intercept) | -0.102 | - | -0.670 | 0.504 | -0.3567, 0.3557 | - |
|  |  | Lockdown_status | 0.248 | - | 2.420 | **0.016** | **0.0830, 0.3802** | - |
|  |  | Human_activity | -0.0001 | - | -1.665 | 0.097 | -0.0001, 0.0000, | - |
|  |  | Noise | 0.020 | - | 8.471 | **< 0.001** | **0.0133, 0.023** | - |
|  |  | Temperature | -0.014 | - | -2.988 | **0.004** | **-0.0274, -0.0074** | - |
|  |  | Lockdown_status*Count_down | -0.002 | - | -1.651 | 0.103 | -0.0041, 0.0005 | - |
|  |  | Lockdown_status*Noise | -0.004 | - | -1.777 | 0.076 | -0.0075, 0.0008 | - |
|  |  | Lockdown_status*Human_activity | -0.0001 | - | -1.498 | 0.135 | -0.0001, 0.00001 | - |
| Graceful  prinia | **^#^Activity-**  Number of  events/day | (Intercept) | 1.536 | 1.139 | - | 0.255 | -1.1049, 4.2863 | - |
|  |  | Lockdown_status | 2.201 | 6.528 | **-** | **< 0.001** | **1.2948, 2.9911** | 83.40 |
|  |  | Human_activity | -0.0001 | -1.481 | - | 0.139 | -0.0003, 0.00004 | 0.01 |
|  |  | Noise | -0.148 | -21.105 | **-** | **< 0.001** | **-0.1657, -0.1326** | 13.76 |
|  |  | Temperature | 0.416 | 7.606 | **-** | **< 0.001** | **0.3083, 0.5235** | 51.59 |
|  |  | Site_category_residence | -1.126 | -1.710 | - | 0.087 | -2.4253, 0.1648 | 67.57 |
|  |  | Site_category_road | -1.519 | -2.368 | **-** | **0.018** | **-2.7761, -0.2513** | 78.11 |
|  |  | Lockdown_status*Count_down | -0.077 | -9.716 | **-** | **< 0.001** | **-0.0919, -0.0609** | 7.41 |
|  |  | Lockdown_status*Site_category_residence | -0.163 | -3.044 | **-** | **0.002** | **-0.2748, -0.0410** | 15.04 |
|  |  | Lockdown_status*Site_category_road | -0.909 | -11.494 | **-** | **< 0.001** | **-1.0850, -0.7472** | 59.71 |
|  |  | Lockdown_status*Human_activity | 0.001 | 8.404 | **-** | **< 0.001** | **0.0005, 0.0008** | 0.10 |
|  | **^#^Activity**  **Variability-**  CV of the  number of  events/day | (Intercept) | 1.353 | - | 2.276 | 0.024 | -0.4557, 2.6207 | - |
|  |  | Lockdown_status | -0.482 | - | -3.197 | **0.002** | **-1.0859, 0.4182** | - |
|  |  | Noise | 0.025 | - | 2.800 | **0.006** | **0.0049, 0.0461** | - |
|  |  | Temperature | -0.072 | - | -3.485 | **0.001** | **-0.1125, -0.0119** | - |
|  |  | Site_category_residence | 0.208 | - | 0.990 | 0.331 | -0.1579, 0.6552 | - |
|  |  | Site_category_road | 0.415 | - | 2.000 | 0.055 | 0.0730, 0.9539 | - |
|  |  | Lockdown_status*Count_down | 0.011 | - | 2.399 | **0.017** | **0.0012, 0.0199** | - |
|  |  | Lockdown_status*Site_category_residence | 0.124 | - | 0.976 | 0.330 | -0.1430, 0.3940 | - |
|  |  | Lockdown_status*Site_category_road | 0.308 | - | 2.157 | **0.032** | **0.0249, 0.6052** | - |

CV: coefficient of variance. *: Interaction effect. 95% confidence intervals of the parameters that did not overlap zero are indicated in bold. **#**: model average.

**Supplementary file 1o.** **Akaike’s information criterion (AICc) model comparison results for activity and activity variability in all species and each bird species.**

| Species | Model description | *df* | logLik | AICc | ∆AICc | *wi* |
| --- | --- | --- | --- | --- | --- | --- |
| **All species** | **Activity (Number of events/day)** ~ |  |  |  |  |  |
|  | Bird, Lock, Noise, Site, Human, Temperature, Bird*Lock, Count*Lock, Lock*Noise, Lock*Site, Lock*Human | 16 | -24379.21 | 48790.83 | 0.00 | 0.98 |
|  | Bird, Lock, Noise, Site, Human, Temperature, Bird*Lock, Noise*Lock, Lock*Site, Lock*Human | 15 | -24384.24 | 48798.84 | 8.01 | 0.02 |
| **All species** | **Activity Variability (CV of the number of events/day) ~** |  |  |  |  |  |
|  | Bird, Lock, Noise, Site, Human, Temperature, Bird*Lock, Lock*Noise | 13 | 215.89 | -405.48 | 0.00 | 0.21 |
|  | Bird, Lock, Noise, Site, Human, Temperature, Bird*Lock, Lock*Noise, Lock*Human | 14 | 216.37 | -404.40 | 1.08 | 0.12 |
|  | Bird, Lock, Noise, Site, Human, Temperature, Bird*Lock, Lock*Noise, Lock*Site | 15 | 217.39 | -404.38 | 1.10 | 0.12 |
|  | Bird, Lock, Noise, Site, Human, Temperature, Bird*Lock, Count*Lock, Lock*Noise | 14 | 215.95 | -403.55 | 1.93 | 0.08 |
|  | Bird, Lock, Noise, Site, Temperature, Bird*Lock, Lock*Noise | 12 | 213.89 | -403.54 | 1.95 | 0.08 |
|  | Bird, Lock, Noise, Site, Human, Temperature, Bird*Lock | 12 | 213.70 | -403.15 | 2.33 | 0.07 |
|  | Bird, Lock, Noise, Site, Human, Temperature, Bird*Lock, Count*Lock, Lock*Noise, Lock*Site | 16 | 217.52 | -402.60 | 2.88 | 0.05 |
|  | Bird, Lock, Noise, Site, Temperature, Bird*Lock | 11 | 212.40 | -402.58 | 2.90 | 0.05 |
|  | Bird, Lock, Noise, Site, Human, Temperature, Bird*Lock, Lock*Site | 14 | 215.43 | -402.53 | 2.95 | 0.05 |
|  | Bird, Lock, Noise, Site, Human, Temperature, Bird*Lock, Count*Lock, Lock*Noise, Lock*Human | 15 | 216.45 | -402.51 | 2.97 | 0.05 |
|  | Bird, Lock, Noise, Site, Human, Temperature, Bird*Lock, Lock*Noise, Lock*Site, Lock*Human | 16 | 217.45 | -402.46 | 3.02 | 0.05 |
|  | Bird, Lock, Noise, Site, Human, Temperature, Bird*Lock, Lock*Human | 13 | 214.26 | -402.22 | 3.26 | 0.04 |
|  | Bird, Lock, Noise, Site, Temperature, Bird*Lock, Count*Lock, Lock*Noise | 13 | 213.94 | -401.58 | 3.90 | 0.03 |
| **Hooded crow** | **Activity (Number of events/day)** ~ |  |  |  |  |  |
|  | Lock, Noise, Site, Human, Count*Lock, Lock*Noise, Lock*Site, Lock*Human | 13 | -4410.91 | 8848.64 | 0.00 | 0.70 |
|  | Lock, Noise, Site, Human, Temperature, Count*Lock, Lock*Noise, Lock*Site, Lock*Human | 14 | -4410.76 | 8850.47 | 1.83 | 0.28 |
|  | Lock, Noise, Site, Human, Count*Lock, Lock*Site, Lock*Human | 12 | -4416.08 | 8856.87 | 8.22 | 0.01 |
|  | Lock, Noise, Site, Human, Temperature, Count*Lock, Lock*Site, Lock*Human | 13 | -4415.58 | 8858.00 | 9.35 | 0.01 |
| **Hooded crow** | **Activity Variability (CV of the number of events/day) ~** |  |  |  |  |  |
|  | Lock, Noise, Site, Human, Temperature, Lock*Noise, Lock*Human | 12 | 213.04 | -401.37 | 0.00 | 0.26 |
|  | Lock, Noise, Site, Human, Lock*Noise, Lock*Human | 11 | 211.47 | -400.35 | 1.03 | 0.16 |
|  | Lock, Noise, Site, Human, Count*Lock, Lock*Noise, Lock*Human | 12 | 212.22 | -399.74 | 1.64 | 0.11 |
|  | Lock, Noise, Site, Human, Temperature, Count*Lock, Lock*Noise, Lock*Human | 13 | 213.06 | -399.30 | 2.08 | 0.09 |
|  | Lock, Noise, Site, Human, Temperature, Lock*Noise, Lock*Site, Lock*Human | 14 | 213.93 | -398.90 | 2.47 | 0.08 |
|  | Lock, Noise, Site, Human, Temperature, Lock*Noise, Lock*Site | 13 | 212.52 | -398.22 | 3.16 | 0.05 |
|  | Lock, Noise, Site, Temperature, Lock*Noise | 10 | 209.26 | -398.03 | 3.35 | 0.05 |
|  | Lock, Noise, Site, Human, Temperature, Lock*Noise | 11 | 210.24 | -397.88 | 3.49 | 0.05 |
|  | Lock, Noise, Site, Human, Lock*Noise, Lock*Site, Lock*Human | 13 | 212.32 | -397.81 | 3.56 | 0.04 |
|  | Lock, Noise, Site, Lock*Noise | 9 | 208.01 | -397.61 | 3.76 | 0.04 |
|  | Lock, Noise, Site, Human, Lock*Noise, Lock*Site | 12 | 211.10 | -397.48 | 3.89 | 0.04 |
|  | Lock, Noise, Human, Temperature, Lock*Noise, Lock*Human | 10 | 208.94 | -397.38 | 3.99 | 0.04 |
| **Rose-ringed**  **parakeet** | **Activity (Number of events/day)** ~ |  |  |  |  |  |
|  | Lock, Noise, Site, Human, Temperature, Lock*Noise, Lock*Site, Lock*Human | 13 | -3029.05 | 6084.94 | 0.00 | 0.74 |
|  | Lock, Noise, Site, Human, Temperature, Count*Lock, Lock*Noise, Lock*Site, Lock*Human | 14 | -3029.05 | 6087.05 | 2.12 | 0.26 |
| **Rose-ringed**  **parakeet** | **Activity Variability (CV of the number of events/day) ~** |  |  |  |  |  |
|  | Lock, Noise, Human, Temperature, Count*Lock, Lock*Noise, Lock*Human | 11 | 393.36 | -764.11 | 0.00 | 0.08 |
|  | Lock, Noise, Human, Temperature, Count*Lock, Lock*Noise | 10 | 392.25 | -763.99 | 0.12 | 0.08 |
|  | Lock, Noise, Human, Temperature, Lock*Noise, Lock*Human | 10 | 392.03 | -763.55 | 0.56 | 0.06 |
|  | Lock, Noise, Human, Temperature, Lock*Human | 9 | 390.85 | -763.30 | 0.81 | 0.06 |
|  | Noise, Human, Temperature | 7 | 388.76 | -763.27 | 0.84 | 0.05 |
|  | Lock, Noise, Site, Human, Temperature, Count*Lock, Lock*Noise, Lock*Human | 13 | 395.03 | -763.22 | 0.89 | 0.05 |
|  | Lock, Noise, Human, Temperature, Count*Lock, Lock*Noise, Lock*Site, Lock*Human | 10 | 391.82 | -763.13 | 0.98 | 0.05 |
|  | Lock, Noise, Site, Human, Temperature, Count*Lock, Lock*Human | 9 | 390.73 | -763.05 | 1.06 | 0.05 |
|  | Lock, Noise, Site, Human, Temperature, Count*Lock, Lock*Noise | 12 | 393.86 | -763.01 | 1.10 | 0.05 |
|  | Lock, Noise, Site, Human, Temperature, Lock*Noise, Lock*Human | 12 | 393.83 | -762.93 | 1.18 | 0.05 |
|  | Lock, Noise, Human, Temperature, Count*Lock | 9 | 390.64 | -762.87 | 1.24 | 0.04 |
|  | Lock, Noise, Site, Human, Temperature, Count*Lock, Lock*Site, Lock*Human | 14 | 395.91 | -762.85 | 1.26 | 0.04 |
|  | Lock, Noise, Human, Temperature | 8 | 389.51 | -762.69 | 1.42 | 0.04 |
|  | Lock, Noise, Site, Human, Temperature, Lock*Human | 11 | 392.64 | -762.67 | 1.44 | 0.04 |
|  | Noise, Site, Human, Temperature | 9 | 390.42 | -762.43 | 1.68 | 0.04 |
|  | Lock, Noise, Site, Human, Temperature, Lock*Site, Lock*Human | 13 | 394.63 | -762.42 | 1.69 | 0.04 |
|  | Lock, Noise, Site, Human, Temperature, Lock*Noise | 11 | 392.47 | -762.33 | 1.78 | 0.03 |
|  | Lock, Noise, Site, Human, Temperature, Count*Lock, Lock*Human | 12 | 393.50 | -762.28 | 1.83 | 0.03 |
|  | Lock, Noise, Site, Human, Temperature | 10 | 391.23 | -761.96 | 2.15 | 0.03 |
|  | Lock, Noise, Site, Human, Temperature, Count*Lock | 11 | 392.26 | -761.91 | 2.20 | 0.03 |
|  | Lock, Noise, Site, Human, Temperature, Count*Lock, Lock*Noise, Lock*Site, Lock*Human | 15 | 396.35 | -761.58 | 2.53 | 0.02 |
|  | Lock, Noise, Site, Human, Temperature, Lock*Noise, Lock*Site, Lock*Human | 14 | 395.04 | -761.10 | 3.01 | 0.02 |
|  | Lock, Noise, Site, Human, Temperature, Count*Lock, Lock*Site | 13 | 393.67 | -760.49 | 3.62 | 0.01 |
| **Graceful prinia** | **Activity (Number of events/day)** ~ |  |  |  |  |  |
|  | Lock, Noise, Site, Human, Temperature, Count*Lock, Lock*Site, Lock*Human | 13 | -6704.26 | 13435.34 | 0.00 | 0.72 |
|  | Lock, Noise, Site, Human, Temperature, Count*Lock, Lock*Noise, Lock*Site, Lock*Human | 14 | -6704.16 | 13437.28 | 1.94 | 0.28 |
| **Graceful prinia** | **Activity Variability (CV of the number of events/day) ~** |  |  |  |  |  |
|  | Lock, Noise, Site, Temperature, Count*Lock, Lock*Site | 12 | -194.55 | 414.03 | 0.00 | 0.25 |
|  | Lock, Noise, Site, Temperature, Count*Lock | 10 | -196.89 | 414.44 | 0.41 | 0.20 |
|  | Lock, Noise, Site, Temperature, Count*Lock, Lock*Noise, Lock*Site | 13 | -194.44 | 415.97 | 1.94 | 0.09 |
|  | Lock, Noise, Site, Human, Temperature, Count*Lock, Lock*Site | 13 | -194.49 | 416.07 | 2.04 | 0.09 |
|  | Lock, Noise, Site, Temperature, Count*Lock, Lock*Noise | 11 | -196.82 | 416.42 | 2.39 | 0.08 |
|  | Lock, Noise, Site, Human, Temperature, Count*Lock | 11 | -196.88 | 416.56 | 2.52 | 0.07 |
|  | Noise, Site, Temperature | 8 | -200.33 | 417.09 | 3.06 | 0.05 |
|  | Lock, Noise, Site, Temperature | 9 | -199.31 | 417.16 | 3.13 | 0.05 |
|  | Lock, Noise, Site, Temperature, Lock*Site | 11 | -197.40 | 417.59 | 3.56 | 0.04 |
|  | Lock, Noise, Site, Human, Temperature, Count*Lock, Lock*Noise, Lock*Site | 14 | -194.35 | 417.97 | 3.94 | 0.03 |
|  | Lock, Noise, Site, Human, Temperature, Count*Lock, Lock*Site, Lock*Human | 14 | -194.38 | 418.03 | 4.00 | 0.03 |

Bird: Bird species. Lock: Lockdown status. Site: Site category. Human: Human activity. Count: Count down. AICc: Akaike’s information criterion corrected for small sample size. ΔAICc: the difference between the alternative model and best-fitting model. Models are ranked based on the AICc values from the best to the worst model. *: Interaction effect.

**Supplementary file 1p. The sampling time and lockdown status for each site.**

| Time | Site3 | Site4 | Site5 | Site6 | Site7 | Site8 | Site9 | Site10 | Site11 | Site12 | Site13 | Site14 | Site15 | Site16 | Site17 | Site18 | Site19 | Lockdown type |
| --- | --- | --- | --- | --- | --- | --- | --- | --- | --- | --- | --- | --- | --- | --- | --- | --- | --- | --- |
| 2020/3/25 | √ | √ | √ | √ | √ | √ | √ | √ |  |  |  |  |  |  |  |  |  | lockdown |
| 2020/3/26 | √ | √ | √ | √ | √ | √ | √ | √ |  |  |  |  |  |  |  |  |  | lockdown |
| 2020/3/27 | √ | √ | √ | √ | √ | √ | √ | √ |  |  |  |  |  |  |  |  |  | lockdown |
| 2020/3/28 | √ | √ | √ | √ | √ | √ | √ | √ |  |  |  |  |  |  |  |  |  | lockdown |
| 2020/3/30 | √ | √ | √ | √ | √ | √ | √ | √ |  |  |  |  |  |  |  |  |  | lockdown |
| 2020/3/31 | √ | √ | √ | √ | √ | √ | √ | √ | √ | √ | √ | √ | √ | √ | √ | √ | √ | lockdown |
| 2020/4/1 | √ | √ | √ | √ | √ | √ | √ | √ | √ | √ | √ | √ | √ | √ | √ | √ | √ | lockdown |
| 2020/4/2 | √ | √ | √ | √ | √ | √ | √ | √ | √ | √ | √ | √ | √ | √ | √ | √ | √ | lockdown |
| 2020/4/3 | √ | √ | √ | √ | √ | √ | √ | √ | √ | √ | √ | √ | √ | √ | √ | √ | √ | lockdown |
| 2020/4/4 | √ | √ | √ | √ | √ | √ | √ | √ | √ | √ | √ | √ | √ | √ | √ | √ | √ | lockdown |
| 2020/4/5 | √ | √ | √ | √ | √ | √ | √ | √ | √ | √ | √ | √ | √ | √ | √ | √ | √ | lockdown |
| 2020/4/6 | √ | √ | √ | √ | √ | √ | √ | √ | √ | √ | √ | √ | √ | √ | √ | √ | √ | lockdown |
| 2020/4/7 | √ | √ | √ | √ | √ | √ | √ | √ | √ | √ | √ | √ | √ | √ | √ | √ | √ | lockdown |
| 2020/4/8 | √ | √ | √ | √ | √ | √ | √ |  | √ | √ | √ | √ | √ | √ | √ | √ | √ | lockdown |
| 2020/4/9 |  |  |  |  |  |  |  |  |  | √ | √ |  |  |  |  |  |  | lockdown |
| 2020/4/24 |  | √ | √ | √ |  | √ | √ | √ | √ | √ |  |  |  | √ |  |  |  | lockdown |
| 2020/4/25 |  | √ | √ | √ |  | √ | √ | √ | √ | √ |  |  |  | √ |  |  |  | lockdown |
| 2020/4/26 |  | √ | √ | √ |  | √ | √ | √ | √ | √ |  |  |  | √ |  |  |  | lockdown |
| 2020/4/27 |  | √ | √ | √ |  | √ | √ | √ | √ | √ |  |  |  | √ |  |  |  | lockdown |
| 2020/4/28 |  | √ | √ | √ |  | √ | √ | √ | √ | √ |  |  |  | √ |  |  |  | lockdown |
| 2020/4/29 |  | √ | √ | √ |  | √ | √ | √ | √ | √ |  |  |  | √ |  |  |  | lockdown |
| 2020/4/30 |  | √ | √ | √ |  | √ | √ | √ | √ | √ |  |  |  | √ |  |  |  | lockdown |
| 2020/5/1 |  | √ | √ | √ |  | √ | √ | √ | √ | √ |  |  |  | √ |  |  |  | lockdown |
| 2020/5/2 |  | √ | √ | √ |  | √ | √ | √ | √ | √ |  |  |  | √ |  |  |  | lockdown |
| 2020/5/3 |  |  | √ | √ |  | √ | √ |  |  |  |  |  |  | √ |  |  |  | lockdown |
| 2020/5/7 |  | √ | √ | √ |  | √ | √ | √ | √ | √ |  |  |  | √ |  | √ |  | lockdown |
| 2020/5/8 |  | √ | √ | √ |  | √ | √ | √ | √ | √ |  |  |  | √ |  | √ |  | lockdown |
| 2020/5/9 |  | √ | √ | √ |  | √ | √ | √ | √ | √ |  |  |  | √ |  | √ |  | lockdown |
| 2020/5/10 |  | √ | √ | √ |  | √ | √ | √ | √ | √ |  |  |  | √ |  | √ |  | lockdown |
| 2020/5/11 |  | √ | √ | √ |  | √ | √ | √ | √ | √ |  |  |  | √ |  | √ |  | lockdown |
| 2020/5/12 |  | √ | √ | √ |  | √ | √ | √ | √ | √ |  |  |  | √ |  | √ |  | lockdown |
| 2020/5/13 |  | √ | √ | √ |  | √ | √ | √ | √ | √ |  |  |  | √ |  | √ |  | lockdown |
| 2020/5/14 |  | √ | √ | √ |  | √ | √ | √ | √ | √ |  |  |  | √ |  | √ |  | lockdown |
| 2020/5/15 |  | √ | √ | √ |  | √ | √ | √ | √ | √ |  |  |  | √ |  | √ |  | lockdown |
| 2020/5/16 |  | √ | √ | √ |  | √ | √ | √ | √ | √ |  |  |  | √ |  | √ |  | lockdown |
| 2020/5/21 | √ | √ | √ | √ | √ | √ | √ | √ | √ | √ | √ | √ | √ | √ | √ | √ | √ | No lockdown |
| 2020/5/22 | √ | √ | √ | √ | √ | √ | √ | √ | √ | √ | √ | √ | √ | √ | √ | √ | √ | No lockdown |
| 2020/5/23 | √ | √ | √ | √ | √ | √ | √ | √ | √ | √ | √ | √ | √ | √ | √ | √ | √ | No lockdown |
| 2020/5/24 | √ | √ | √ | √ | √ | √ | √ | √ | √ | √ | √ | √ | √ | √ | √ | √ | √ | No lockdown |
| 2020/5/25 | √ | √ | √ | √ | √ | √ | √ | √ | √ | √ | √ | √ | √ | √ | √ | √ | √ | No lockdown |
| 2020/5/26 | √ | √ | √ | √ | √ | √ | √ | √ | √ | √ | √ | √ | √ | √ | √ | √ | √ | No lockdown |
| 2020/5/27 | √ | √ | √ | √ | √ | √ | √ | √ | √ | √ | √ | √ | √ | √ | √ | √ | √ | No lockdown |
| 2020/5/28 | √ | √ | √ | √ | √ | √ | √ | √ | √ | √ | √ | √ | √ | √ | √ | √ | √ | No lockdown |
| 2020/5/29 | √ | √ | √ |  | √ | √ | √ | √ |  | √ | √ | √ | √ | √ | √ | √ | √ | No lockdown |
| 2020/5/30 |  | √ |  |  |  |  |  |  |  |  | √ | √ | √ | √ | √ | √ | √ | No lockdown |

Note: "√" means there are audio files

**Supplementary file 1q. The latitude and longitude of the sampling site.**

| Site | Latitude (°N) | Longitude (°E) |
| --- | --- | --- |
| Site3 | 32.095132 | 34.788665 |
| Site4 | 32.095708 | 34.793194 |
| Site5 | 32.093243 | 34.793475 |
| Site6 | 32.09152 | 34.788699 |
| Site7 | 32.093405 | 34.784918 |
| Site8 | 32.089411 | 34.785112 |
| Site9 | 32.090122 | 34.792359 |
| Site10 | 32.088949 | 34.790817 |
| Site11 | 32.096744 | 34.789951 |
| Site12 | 32.095503 | 34.783913 |
| Site13 | 32.094899 | 34.792372 |
| Site14 | 32.093886 | 34.789753 |
| Site15 | 32.091865 | 34.791897 |
| Site16 | 32.09169 | 34.78334 |
| Site17 | 32.091016 | 34.784929 |
| Site18 | 32.091107 | 34.789882 |
| Site19 | 32.089007 | 34.787887 |

**Supplementary file 1r.** **Effects of predictor variables on birds’ activity based on generalized and general linear mixed models (GLMM and LMM). Estimates were calculated in % per day for the following units: Temperature – per degree, Noise – per dB, Human activity – per 1 talking event, Lockdown related parameter – per existence of the lockdown (yes/no).**

| Species | Dependent  variable | Predictors | Estimate | *z* | t | *p* | 95% CI | Percent |
| --- | --- | --- | --- | --- | --- | --- | --- | --- |
| All species | **Activity-**  Number of  events/day | (Intercept) | 7.555 | 22.048 | - | **<** 0.001 | - | - |
|  |  | Bird_species | -0.623 | -84.742 | - | **< 0.001** | - | 46.367 |
|  |  | Lockdown_status | -0.064 | -0.437 | - | 0.662 | - | 6.200 |
|  |  | Human_activity | -0.00002 | -0.725 | - | 0.469 | - | 0.002 |
|  |  | Noise | -0.062 | -21.471 | - | **< 0.001** | - | 6.012 |
|  |  | Temperature | 0.055 | 5.114 | - | **< 0.001** | - | 5.711 |
|  |  | Site_category_residence | -0.762 | -2.927 | - | **0.003** | - | 54.393 |
|  |  | Site_category_road | -0.917 | -3.626 | - | **<0.001** | - | 61.829 |
|  |  | Lockdown_status*Count_down | 0.005 | 2.552 | - | **0.011** | - | 0.521 |
|  |  | Lockdown_status*Site_category_residence | -0.068 | -3.213 | - | **0.001** | - | 6.903 |
|  |  | Lockdown_status*Site_category_road | 0.068 | 3.003 | - | **0.003** | - | 7.459 |
|  |  | Lockdown_status*Noise | -0.014 | -5.400 | - | **< 0.001** | - | 1.488 |
|  |  | Lockdown_status*Human_activity | 0.0004 | 14.825 | - | **< 0.001** | - | 0.043 |
|  |  | Bird_species*Lockdown_status | 0.253 | 29.155 | - | **< 0.001** | - | 31.379 |
|  | **^#^Activity**  **Variability-**  CV of the  number of  events/day | (Intercept) | -0.835 | - | -5.908 | < 0.001 | -1.167, -0.527 | - |
|  |  | Bird_species | 0.208 | - | 16.856 | **< 0.001** | **0.183, 0.232** | - |
|  |  | Lockdown_status | 0.466 | - | 3.946 | **< 0.001** | **0.157, 0.746** | - |
|  |  | Human_activity | -0.00005 | - | -2.224 | **0.026** | **-0.0001, -0.00002** | - |
|  |  | Noise | 0.024 | - | 9.033 | **< 0.001** | **0.018, 0.029** | - |
|  |  | Temperature | -0.012 | - | -4.571 | **< 0.001** | **-0.017, -0.0052** | - |
|  |  | Site_category_residence | 0.175 | - | 2.546 | **0.021** | **0.035, 0.313** | - |
|  |  | Site_category_road | 0.232 | - | 3.471 | **0.003** | **0.093, 0.363** | - |
|  |  | Lockdown_status*Noise | -0.007 | - | -2.908 | **0.004** | **-0.012, -0.002** | - |
|  |  | Bird_species*Lockdown_status | -0.056 | - | -3.838 | **< 0.001** | **-0.085, -0.027** | - |
| Hooded  crow | **^#^Activity-**  Number of  events/day | (Intercept) | 6.901 | 22.934 | - | < 0.001 | 6.165, 7.608 | - |
|  |  | Lockdown_status | -0.627 | -7.950 | - | **< 0.001** | **-0.887, -0.169** | 46.581 |
|  |  | Human_activity | -0.0001 | -3.540 | - | **< 0.001** | **-0.0002, -0.00005** | 0.010 |
|  |  | Noise | -0.036 | -11.648 | - | **< 0.001** | **-0.042, -0.027** | 3.536 |
|  |  | Site_category_residence | -0.756 | -2.294 | - | **0.022** | **-1.399, -0.102** | 53.046 |
|  |  | Site_category_road | -0.773 | -2.413 | - | **0.016** | **-1.408, -0.149** | 54.376 |
|  |  | Count_down*Lockdown_status | 0.015 | 6.235 | - | **< 0.001** | **0.010, 0.020** | 1.542 |
|  |  | Lockdown_status*Site_category_residence | -0.064 | -2.399 | - | **0.017** | **-0.132, -0.015** | 6.385 |
|  |  | Lockdown_status*Site_category_road | 0.193 | 6.934 | - | **< 0.001** | **0.143, 0.261** | 22.140 |
|  |  | Lockdown_status*Human_activity | 0.0003 | 9.935 | - | **< 0.001** | **0.0003, 0.0004** | 0.032 |
|  | **^#^Activity**  **ariability-**  CV of the  number of  events/day | (Intercept) | -1.161 | - | -7.446 | **< 0.001** | **-1.662,-0.882** | - |
|  |  | Lockdown_type | 0.841 | - | 6.346 | **< 0.001** | **0.450, 1.122** | - |
|  |  | Human_activity | 0.00004 | - | 0.999 | 0.318 | -0.00001, 0.0001 | - |
|  |  | Noise | 0.032 | - | 10.567 | **< 0.001** | **0.025, 0.038** | - |
|  |  | Count_down*Lockdown_status | -0.001 | - | -1.143 | 0.256 | **-0.003, 0.001** | - |
|  |  | Lockdown_status*Noise | -0.014 | - | -5.163 | **< 0.001** | **-0.020, -0.007** | - |
|  |  | Lockdown_status*Human_activity | -0.0001 | - | -2.717 | **0.007** | **-0.0002, -0.00003** | - |
| Rose-ringed  parakeet | **^#^Activity-**  Number of  events/day | (Intercept) | 6.152 | 12.169 | - | < 0.001 | 5.206, 7.255 | - |
|  |  | Lockdown_type | -0.051 | -0.213 | - | 0.832 | -0.604, 0.408 | 4.972 |
|  |  | Human_activity | 0.0003 | -6.909 | - | **< 0.001** | **-0.0004, -0.0002** | 0.030 |
|  |  | Noise | -0.063 | -11.621 | - | **< 0.001** | **-0.074, -0.052** | 6.106 |
|  |  | Temperature | 0.068 | 4.838 | - | **< 0.001** | **0.035, 0.094** | 7.037 |
|  |  | Site_category_residence | -0.522 | -1.267 | - | 0.205 | -1.330, 0.290 | 41.073 |
|  |  | Site_category_road | -1.189 | -2.972 | - | **0.003** | **-1.975, -0.400** | 70.938 |
|  |  | Lockdown_status*Site_category_residence | 0.430 | 10.786 | - | **< 0.001** | **0.344, 0.506** | 55.338 |
|  |  | Lockdown_status*Site_category_road | 0.523 | 12.416 | - | **< 0.001** | **0.441, 0.607** | 71.456 |
|  |  | Lockdown_status*Noise | -0.017 | -3.563 | - | **< 0.001** | **-0.026, -0.007** | 1.770 |
|  |  | Lockdown_status*Human_activity | 0.001 | 15.772 | - | **< 0.001** | **0.0006, 0.0008** | 0.106 |
|  | **^#^Activity**  **ariability-**  CV of the  number of  events/day | (Intercept) | -0.316 | - | -2.604 | 0.010 | -0.630, -0.075 | - |
|  |  | Lockdown_status | 0.200 | - | 5.873 | **< 0.001** | **0.047, 0.439** | - |
|  |  | Noise | 0.018 | - | 9.637 | **< 0.001** | **0.014, 0.024** | - |
|  |  | Site_category_residence | 0.087 | - | 1.035 | 0.314 | -0.079, 0.254 | - |
|  |  | Site_category_road | 0.201 | - | 2.467 | **0.023** | **0.034, 0.358** | - |
|  |  | Human_activity | 0.00001 | - | 0.301 | 0.763 | -0.0001, 0.0001 | - |
|  |  | Temperature | -0.008 | - | -2.608 | **0.013** | **-0.013, -0.002** | - |
|  |  | Count_down*Lockdown_status | -0.003 | - | -4.162 | **< 0.001** | **-0.005, -0.002** | - |
|  |  | Lockdown_status*Site_category_residence | -0.050 | - | -1.867 | 0.063 | -0.109, 0.004 | - |
|  |  | Lockdown_status*Site_category_road | -0.104 | - | -3.903 | **< 0.001** | **-0.156, -0.043** | - |
|  |  | Lockdown_status*Human_activity | -0.00001 | - | -2.811 | **0.005** | **-0.0002, -0.00002** | - |
| Graceful  prinia | **Activity-**  Number of  events/day | (Intercept) | 4.013 | 2.593 | - | 0.010 | - | - |
|  |  | Lockdown_status | 1.008 | 1.714 | - | 0.086 | - | 174.012 |
|  |  | Human_activity | 0.0001 | 1.984 | - | 0.047 | - | 0.010 |
|  |  | Noise | -0.198 | -20.677 | - | **< 0.001** | - | 17.963 |
|  |  | Temperature | 0.395 | 6.631 | - | **< 0.001** | - | 48.438 |
|  |  | Site_category_residence | -1.254 | -1.797 | - | 0.072 | - | 72.179 |
|  |  | Site_category_road | -1.424 | -2.093 | - | 0.036 | - | 77.444 |
|  |  | Lockdown_status*Count_down | -0.105 | -15.002 | - | **< 0.001** | - | 10.267 |
|  |  | Lockdown_status*Site_category_residence | -0.041 | -0.744 | - | 0.457 | - | 4.178 |
|  |  | Lockdown_status*Site_category_road | -1.036 | -11.776 | - | **< 0.001** | - | 67.739 |
|  |  | Lockdown_status*Noise | 0.035 | 3.898 | - | **< 0.001** | - | 3.776 |
|  |  | Lockdown_status*Human_activity | 0.0005 | 6.357 | - | **< 0.001** | - | 0.054 |
|  | **^#^Activity**  **ariability-**  CV of the  number of  events/day | (Intercept) | 0.698 | - | 1.548 | 0.123 | -0.472, 1.608 | - |
|  |  | Lockdown_status | -0.324 | - | -2.978 | **0.003** | -0.824, 0.361 | - |
|  |  | Noise | 0.024 | - | 2.903 | **0.004** | **0.006, 0.043** | - |
|  |  | Temperature | -0.040 | - | -3.538 | **0.005** | **-0.059, -0.012** | - |
|  |  | Site_category_residence | 0.211 | - | 0.983 | 0.336 | -0.165, 0.680 | - |
|  |  | Site_category_road | 0.432 | - | 2.036 | 0.052 | **0.078, 0.961** | - |
|  |  | Count_down*Lockdown_status | 0.005 | - | 1.608 | 0.109 | -0.001, 0.011 | - |
|  |  | Lockdown_status*Site_category_residence | 0.146 | - | 1.316 | 0.189 | -0.079, 0.382 | - |
|  |  | Lockdown_status*Site_category_road | 0.285 | - | 2.245 | **0.025** | **0.025, 0.536** | - |

CV: coefficient of variance. *: Interaction effect. 95% confidence intervals of the parameters that did not overlap zero are indicated in bold. **#**: model average.

**Supplementary file 1s. Akaike’s information criterion (AICc) model comparison results for activity and activity variability in all species and each bird species.**

| Species | Model description | *df* | logLik | AICc | ∆AICc | *wi* |
| --- | --- | --- | --- | --- | --- | --- |
| **All species** | **Activity (Number of events/day)** ~ |  |  |  |  |  |
|  | Bird, Lock, Noise, Site, Human, Temperature, Bird*Lock, Count*Lock, Lock*Noise, Lock*Site, Lock*Human | 16 | -29663.59 | 59359.52 | 0.00 | 0.90 |
|  | Bird, Lock, Noise, Site, Human, Temperature, Bird*Lock, Noise*Lock, Lock*Site, Lock*Human | 15 | -29666.80 | 59363.90 | 4.38 | 0.10 |
| **All species** | **Activity Variability (CV of the number of events/day) ~** |  |  |  |  |  |
|  | Bird, Lock, Noise, Site, Human, Temperature, Bird*Lock, Lock*Noise | 13 | 237.72 | -449.20 | 0.00 | 0.23 |
|  | Bird, Lock, Noise, Site, Human, Temperature, Bird*Lock, Count*Lock, Lock*Noise | 14 | 238.72 | -449.15 | 0.05 | 0.22 |
|  | Bird, Lock, Noise, Site, Human, Temperature, Bird*Lock, Lock*Noise, Lock*Human | 14 | 238.35 | -448.41 | 0.79 | 0.16 |
|  | Bird, Lock, Noise, Site, Human, Temperature, Bird*Lock, Count*Lock, Lock*Noise, Lock*Human | 15 | 239.35 | -448.38 | 0.82 | 0.15 |
|  | Bird, Lock, Noise, Site, Temperature, Bird*Lock, Count*Lock, Lock*Noise | 13 | 236.51 | -446.77 | 2.42 | 0.07 |
|  | Bird, Lock, Noise, Site, Human, Temperature, Bird*Lock, Lock*Noise, Lock*Site | 15 | 238.46 | -446.60 | 2.60 | 0.06 |
|  | Bird, Lock, Noise, Site, , Temperature, Bird*Lock, Lock*Noise | 12 | 235.30 | -446.38 | 2.82 | 0.06 |
|  | Bird, Lock, Noise, Site, Human, Temperature, Bird*Lock, Count*Lock, Lock*Noise, Lock*Site | 16 | 239.24 | -446.11 | 3.09 | 0.05 |
| **Hooded crow** | **Activity (Number of events/day)** ~ |  |  |  |  |  |
|  | Lock, Noise, Site, Human, Count*Lock, Lock*Site, Lock*Human | 12 | -5349.02 | 10722.64 | 0.00 | 0.36 |
|  | Lock, Noise, Site, Human, Count*Lock, Lock*Noise, Lock*Site, Lock*Human | 13 | -5348.03 | 10722.76 | 0.12 | 0.34 |
|  | Lock, Noise, Site, Human, Temperature, Count*Lock, Lock*Site, Lock*Human | 13 | -5348.81 | 10724.31 | 1.67 | 0.16 |
|  | Lock, Noise, Site, Human, Temperature, Count*Lock, Lock*Noise, Lock*Site, Lock*Human | 14 | -5347.88 | 10724.56 | 1.92 | 0.14 |
| **Hooded crow** | **Activity Variability (CV of the number of events/day) ~** |  |  |  |  |  |
|  | Lock, Noise, Site, Human, Lock*Noise, Lock*Human | 11 | 245.65 | -468.80 | 0.00 | 0.25 |
|  | Lock, Noise, Site, Human, Temperature, Lock*Noise, Lock*Human | 12 | 246.67 | -468.75 | 0.05 | 0.25 |
|  | Lock, Noise, Site, Human, Count*Lock, Lock*Noise, Lock*Human | 12 | 246.26 | -467.93 | 0.87 | 0.16 |
|  | Lock, Noise, Site, Human, Temperature, Count*Lock, Lock*Noise, Lock*Human | 13 | 246.83 | -466.98 | 1.82 | 0.10 |
|  | Lock, Noise, Site, Human, Lock*Noise, Lock*Site, Lock*Human | 13 | 246.28 | -465.87 | 2.93 | 0.06 |
|  | Lock, Noise, Site, Human, Temperature, Lock*Noise, Lock*Site, Lock*Human | 14 | 247.26 | -465.72 | 3.08 | 0.05 |
|  | Lock, Noise, Site, Human, Count*Lock, Lock*Noise, Lock*Site, Lock*Human | 14 | 247.15 | -465.49 | 3.31 | 0.05 |
|  | Lock, Noise, Human, Lock*Noise, Lock*Human | 9 | 241.62 | -464.89 | 3.91 | 0.04 |
|  | Lock, Noise, Human, Temperature, Lock*Noise, Lock*Human | 10 | 242.63 | -464.85 | 3.95 | 0.04 |
| **Rose-ringed**  **parakeet** | **Activity (Number of events/day)** ~ |  |  |  |  |  |
|  | Lock, Noise, Site, Human, Temperature, Lock*Noise, Lock*Site, Lock*Human | 13 | -3599.12 | 7224.94 | 0.00 | 0.59 |
|  | Lock, Noise, Site, Human, Temperature, Count*Lock, Lock*Noise, Lock*Site, Lock*Human | 14 | -3598.44 | 7225.69 | 0.75 | 0.41 |
| **Rose-ringed**  **parakeet** | **Activity Variability (CV of the number of events/day) ~** |  |  |  |  |  |
|  | Lock, Noise, Site, Human, Temperature, Count*Lock, Lock*Site, Lock*Human | 14 | 460.70 | -892.59 | 0.00 | 0.65 |
|  | Lock, Noise, Site, Human, Temperature, Count*Lock, Lock*Noise, Lock*Site, Lock*Human | 15 | 461.16 | -891.39 | 1.20 | 0.35 |
| **Graceful prinia** | **Activity (Number of events/day)** ~ |  |  |  |  |  |
|  | Lock, Noise, Site, Human, Temperature, Count*Lock, Lock*Noise, Lock*Site, Lock*Human | 14 | -7795.18 | 15619.17 | 0.00 | 1.00 |
|  | Lock, Noise, Site, Human, Temperature, Count*Lock, Lock*Site, Lock*Human | 13 | -7802.84 | 15632.38 | 13.21 | 0.00 |
| **Graceful prinia** | **Activity Variability (CV of the number of events/day) ~** |  |  |  |  |  |
|  | Lock, Noise, Site, Temperature, Count*Lock, Lock*Site, Lock*Human | 12 | -225.14 | 475.06 | 0.00 | 0.16 |
|  | Lock, Noise, Site, Temperature, Lock*Site | 11 | -226.43 | 475.52 | 0.45 | 0.12 |
|  | Lock, Noise, Site, Temperature | 9 | -228.62 | 475.69 | 0.63 | 0.11 |
|  | Lock, Noise, Site, Temperature, Count*Lock | 10 | -227.65 | 475.85 | 0.78 | 0.11 |
|  | Noise, Site, Temperature | 8 | -230.19 | 476.74 | 1.68 | 0.07 |
|  | Lock, Noise, Site, Human, Temperature, Count*Lock, Lock*Site | 13 | -225.02 | 476.97 | 1.90 | 0.06 |
|  | Lock, Noise, Site, Temperature, Count*Lock, Lock*Noise, Lock*Site | 13 | -225.12 | 477.17 | 2.10 | 0.05 |
|  | Lock, Noise, Site, Human, Temperature, Lock*Site | 12 | -226.36 | 477.51 | 2.45 | 0.05 |
|  | Lock, Noise, Site, Temperature, Lock*Noise | 10 | -228.53 | 477.62 | 2.55 | 0.04 |
|  | Lock, Noise, Site, Temperature, Lock*Noise, Lock*Site | 12 | -226.42 | 477.63 | 2.57 | 0.04 |
|  | Lock, Noise, Site, Human, Temperature | 10 | -228.62 | 477.80 | 2.73 | 0.04 |
|  | Lock, Noise, Site, Temperature, Count*Lock, Lock*Noise | 11 | -227.59 | 477.85 | 2.78 | 0.04 |
|  | Lock, Noise, Site, Human, Temperature, Count*Lock | 11 | -227.64 | 477.94 | 2.88 | 0.04 |
|  | Noise, Site, Human, Temperature | 9 | -230.19 | 478.83 | 3.77 | 0.02 |
|  | Lock, Noise, Site, Human, Temperature, Count*Lock, Lock*Site, Lock*Human | 14 | -224.92 | 478.91 | 3.85 | 0.02 |
|  | Lock, Noise, Site, Human, Temperature, Count*Lock, Lock*Noise, Lock*Site | 14 | -225.00 | 479.06 | 3.99 | 0.02 |

Bird: Bird species. Lock: Lockdown status. Site: Site category. Human: Human activity. Count: Count down. AICc: Akaike’s information criterion corrected for small sample size. ΔAICc: the difference between the alternative model and best-fitting model. Models are ranked based on the AICc values from the best to the worst model. *: Interaction effect.
